# FAM20C Overview: Classic and Novel Targets, Pathogenic Variants and Raine Syndrome Phenotypes

**DOI:** 10.3390/ijms22158039

**Published:** 2021-07-27

**Authors:** Icela Palma-Lara, Monserrat Pérez-Ramírez, Patricia García Alonso-Themann, Ana María Espinosa-García, Ricardo Godinez-Aguilar, José Bonilla-Delgado, Adolfo López-Ornelas, Georgina Victoria-Acosta, María Guadalupe Olguín-García, José Moreno, Carmen Palacios-Reyes

**Affiliations:** 1 Laboratorio de Morfología Celular y Molecular, Sección de Estudios de Posgrado e Investigación, Instituto Politécnico Nacional, Escuela Superior de Medicina, Ciudad de México 11340, Mexico; icelitpl@yahoo.com (I.P.-L.); monserratpr2006@yahoo.com.mx (M.P.-R.); 2 Instituto Nacional de Perinatología, Seguimiento Pediátrico, Ciudad de México 11000, Mexico; pgalonsot@yahoo.com; 3 Laboratorio de Farmacología Clínica, Hospital General de México, Ciudad de México 06720, Mexico; anaesga@hotmail.com; 4 División de Investigación, Hospital Juárez de México, Ciudad de México 07760, Mexico; raa_rga@yahoo.com.mx (R.G.-A.); jbonilla@cinvestav.mx (J.B.-D.); adolfolopezmd@gmail.com (A.L.-O.); giviac@gmail.com (G.V.-A.); jmoreno49@gmail.com (J.M.); 5 Centro Dermatológico “Dr. Ladislao de la Pascua” SSPCDMX, Departamento de Educación e Investigación, Ciudad de México 06780, Mexico; olguingog@yahoo.com.mx

**Keywords:** pathogenic variants, Raine syndrome, FAM20C targets

## Abstract

*FAM20C* is a gene coding for a protein kinase that targets S-X-E/pS motifs on different phosphoproteins belonging to diverse tissues. Pathogenic variants of *FAM20C* are responsible for Raine syndrome (RS), initially described as a lethal and congenital osteosclerotic dysplasia characterized by generalized atherosclerosis with periosteal bone formation, characteristic facial dysmorphisms and intracerebral calcifications. The aim of this review is to give an overview of targets and variants of FAM20C as well as RS aspects. We performed a wide phenotypic review focusing on clinical aspects and differences between all lethal (LRS) and non-lethal (NLRS) reported cases, besides the *FAM20C* pathogenic variant description for each. As new targets of FAM20C kinase have been identified, we reviewed FAM20C targets and their functions in bone and other tissues, with emphasis on novel targets not previously considered. We found the classic lethal and milder non-lethal phenotypes. The milder phenotype is defined by a large spectrum ranging from osteonecrosis to osteosclerosis with additional congenital defects or intellectual disability in some cases. We discuss our current understanding of FAM20C deficiency, its mechanism in RS through classic FAM20C targets in bone tissue and its potential biological relevance through novel targets in non-bone tissues.

## 1. Introduction

*FAM20C* mutations are the cause of Raine syndrome (RS) (OMIM # 259775) [1], an autosomal recessive lethal disease characterized by generalized osteosclerosis with periosteal bone formation, characteristic facial dysmorphisms and intracerebral calcifications [2]. It has a prevalence of <1 in 1,000,000 [3] and most of those affected die within the first weeks of life due to pulmonary hypoplasia [4,5,6].

Although most cases are detected at birth, facial alterations, such as a flat facial profile, hypoplastic nose and prominent eyes, have been described prenatally, in addition to cerebral alterations, such as large choroid plexuses, an echogenic appearance of the brain, ventricular blurry side walls and intracerebral calcifications [7,8,9,10,11]. The RS phenotype is related to changes in the location of the FAM20C protein and/or its kinase activity [12], caused by different pathogenic variant types. FAM20C targets comprise a broad spectrum of secretory pathway proteins (>100) [13], but mineralization proteins are the most known and understood. However, since 2015, novel FAM20C targets have been described which participate in different biological processes besides bone tissue and, therefore, have a potential effect on its phosphorylation state and function. Due to the wide FAM20C target variability, it is important to consider these targets as potential altered proteins in RS [14,15]

The objective of this review is to give an overview of FAM20C function and RS, including the lethal and non-lethal phenotypes reported to date, and the variants for each type. We also describe the already known FAM20C targets involved in the physiopathology of mineralized tissues such as small integrin-binding ligand N-linked glycoprotein (SIBLING) proteins, but we focus on novel non-mineralized tissue targets not previously considered but with potential biological relevance in RS. We identified a total of 70 cases, 41 lethal and 29 non-lethal, and defined differences between both types, remarking a wide variability in non-lethal cases.

## 2. FAM20 Family

**Figure 1:**
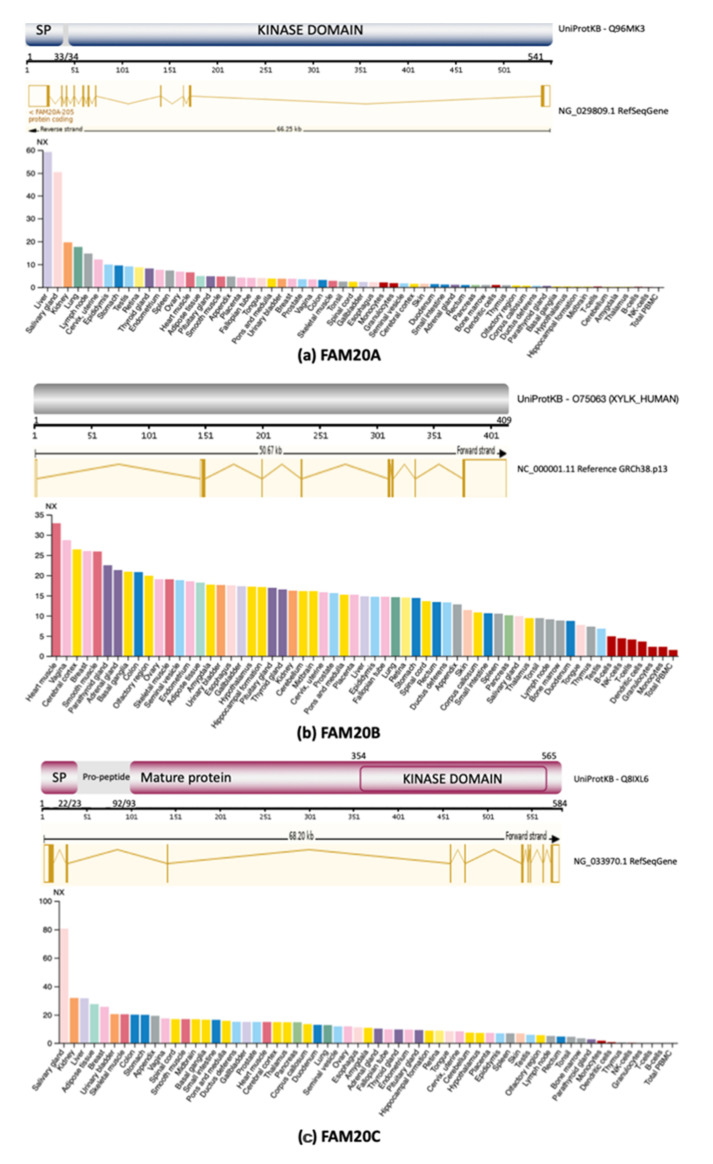
Members of FAM20C family. Protein structure at the top, gene structure in the middle and RNA expression in different tissues in the bottom: (**a**) description of FAM20A; (**b**) description of FAM20B; (**c**) description of FAM20C. Gene structure of each gene was obtained from Ensemble release 104—May 2021 (Access date: 1 June 2021). RNA expression from consensus transcript expression levels from The Human Protein Atlas (https://www.proteinatlas.org/ENSG00000108950-FAM20A/tissue, https://www.proteinatlas.org/ENSG00000116199-FAM20B/tissue, https://www.proteinatlas.org/ENSG00000177706-FAM20C/tissue) version 20.1 and Ensembl version 92.38. NX: consensus normalized expression value for each gene (access date: 15 June 2021).

The FAM20 family was identified from Position-Specific Iterative Basic Local Alignment Search Tool results as a new sequence detected in a hematopoietic stem cell line with similarity to the human four-jointed (fjx1) amino acid sequence, a family of related proteins with essential conserved residues for protein kinase activity, but with weak sequence similarity to canonical protein kinases. Through comparative genomics, three members were identified as belonging to the family named “family with sequence similarity 20” (FAM20), which consists of *FAM20A*, *FAM20B* and *FAM20C* (Figure 1), that encode protein kinases acting on diverse substrates that play important roles in biomineralization [16,17,18]. This family, collectively with O-mannosyl kinase (Sgk196) and the secreted tyrosine kinase vertebrate lonesome kinase (VLK), comprise the secretory pathway kinases that phosphorylate a diverse array of substrates that play important roles in many fundamental physiological processes.

### 2.1. FAM20A

*FAM20A*, mapped at 17q24.2, is considered a pseudokinase because of its lack of activity, but it preserves some conserved sequences (Figure 1a). Pathogenic variants are the cause of entities with enamel defects, including enamel renal syndrome with autosomal recessive inheritance (also called amelogenesis imperfecta type 1G), enamel-renal-gingival syndrome, amelogenesis imperfecta and gingival fibromatosis syndrome (MIM #204 690) [19]. Despite its lack of kinase activity, FAM20A forms a complex with FAM20C that promotes enamel matrix protein phosphorylation. Although this process is not entirely clear, RS features resemble dental phenotypes of FAM20A deficiency, probably due to FAM20A–FAM20C complex disruptions.

### 2.2. FAM20B

*FAM20B*, mapped at 1q25.2, codes for a glycan kinase that phosphorylates the xylose residue in the glycosaminoglycan–protein linkage region (GAG-Xyl kinase) (Figure 1b) [20]. Xylose phosphorylation increases its galactosyltransferase II function during glycosaminoglycan maturation [21,22]. FAM20B deletion is lethal in mice embryo. In humans, only two lethally affected neonatal siblings, with a Desbuquois-like short limb dysplasia, have been related to biallelic pathogenic variants (compound heterozygous truncating variants). Both patients had a very short stature, craniofacial phenotype with a prominent forehead, shallow orbits, depressed nasal bridge, mid-face hypoplasia, protruding eyes, thoracic hypoplasia with respiratory failure, limbs with mesomelic shortening, multiple dislocations and digital aplasia/hypoplasia. Radiological features include prominent lesser trochanter and long bone flared metaphyses without bone density alterations [23]. These loss of function (LoF) protein variants appear to be responsible for the phenotype, as the *XYLY1* gene involved in Desbuquois dysplasia codes for xylosyltransferase-1, an enzyme with a role in proteoglycan synthesis. FAM20B deficiency would provoke hypophosphorylation of galactosyltransferase I, with secondary lower activity on glycosaminoglycan maturation [24]. This defect apparently causes a severe bone dysplasia featured by bone tissue defects and thoracic hypoplasia, as in RS. FAM20B-associated dysplasia has some RS-like features, including mid-face hypoplasia, thoracic hypoplasia, respiratory failure and short limbs, but it lacks ocular proptosis, osteosclerosis and wide fontanelle. In addition, other non-RS features were present as a Desbuquois-like phenotype. Although FAM20B functions as a glycosaminoglycan xylosyl kinase, this case supports some shared functions of FAM20 family members [20,23].

### 2.3. FAM20C

The *FAM20C* gene (*DMP4* in mouse), located at locus 7p22.3, spans 72410pb (NG_033970.1) and contains 10 exons. FAM20C mRNA spans 3176pb (NM_020223.4) and has a 1755 nucleotide code sequence (Figure 1c). It codes for the FAM20C protein, previously known as the Golgi casein kinase (GCK) [25], localized in both the endoplasmic reticulum (ER) and Golgi lumen in lactating breast glands as a 70kDa enzyme that phosphorylates serine residues. It is 584 aa long, and the immature protein has a signal peptide (N-terminal 1-22 aa) that targets the secretory pathway. Its kinase domain (KD) spans 222 aa, from residue 354 to 565 [26].

FAM20C is considered as the archetypical member of the family, recognized mostly as the kinase for mineralization proteins, such as the secretory calcium-binding phosphoprotein (SCCP) protein family and fibroblast growth factor 23 (FGF23). It has ubiquitous expression, especially in mineralized tissues, such as bone, dentine and enamel. Sclerosing bone defects in RS result from mineralization protein hypophosphorylation due to FAM20C deficiency. Moreover, FAM20C is also responsible for generating the majority of the secreted phosphoproteome (>90%). As such, the FAM20C protein is a casein kinase expressed in the Golgi apparatus that phosphorylates serine residues at S-X-E/pS motifs of proteins found in serum, plasma, cerebrospinal fluid and other tissues, estimated to be over 100 genuine substrates [13,17]. It is important to notice that 75% of the phosphoproteins of the cerebrospinal fluid contain the S-x-E/pS motif. Despite the abundant and novel FAM20C targets, hypophosphorylation effects of these novel targets due to FAM20C deficiency in RS remain uncharacterized.

## 3. FAM20C Biological Function in Bone: SIBLING Proteins

**Figure 2:**
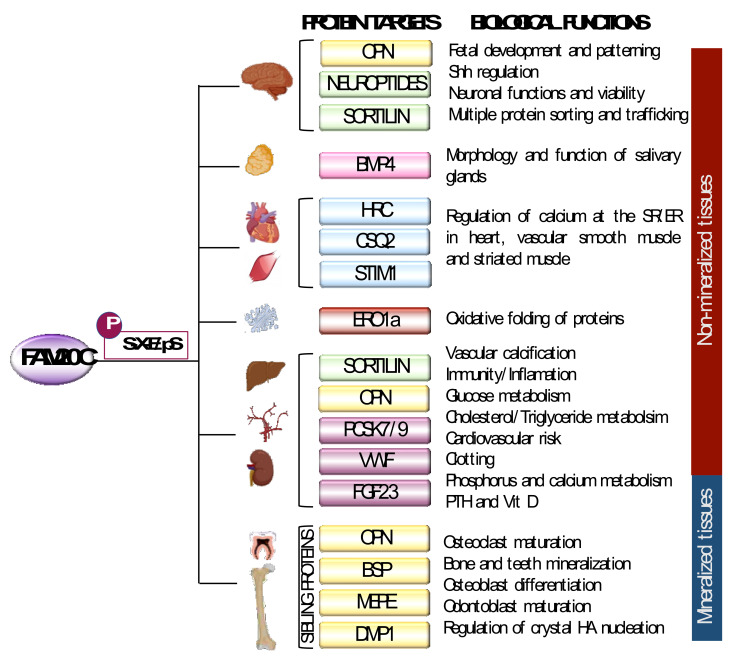
Fam20C classic and novel targets. Picture showing different protein targets of FAM20C and their functions. Classical FAM20C targets with function in mineralization are shown in yellow at the bottom and new described targets are in different colors in the other sections. SR/ER, sarcoplasmic reticulum/endoplasmic reticulum; Vit D, vitamin D; PTH, parathyroid hormone; HA: hydroxyapatite.

FAM20C is involved in the biomineralization process through phosphorylation of members of the secretory calcium-binding phosphoproteins family (non-collagenous proteins) primarily located in bone and dentin. This family spans a 24-gene cluster in chromosome 4q13-q21, which includes five tandem genes that encode the SIBLING proteins, such as dentin matrix protein (DMP1), bone sialoprotein (BSP), osteopontin (OPN), extracellular phosphoglycoprotein matrix (MEPE) and dentine sialophosphoprotein (DSPP) [15,27,28]. These proteins possess high calcium affinity, regulate calcium phosphate precipitation as hydroxyapatite (HA) and are featured by an acidic serine aspartate-rich MEPE-associated motif (ASARM) and an Arg-Gly-Asp (RGD) sequence, as well as multiple S-x-E/pS phosphorylation motifs and roles in HA formation in bone and teeth (Figure 2) [29].

### 3.1. OPN

OPN is a multifunctional and ubiquitously expressed highly phosphorylated protein produced by osteoblasts, osteoclasts, osteocytes and odontoblasts in mineralized tissues. OPN is the main nucleator of mineral in bone because it is able to regulate crystal HA composition and growth and is produced by calcitriol signals, as well as magnesium and phosphate elevation [30,31]. This is achieved through binding to extracellular calcium through its acidic and phosphorylated residues (sites of Ser/Thr HA) on the ASARM motif, thus inhibiting HA formation and growth. OPN has 29 potential phosphorylation sites and its phosphorylation varies in different tissues. Phosphorylated sites slightly modify OPN conformation bound to HA. In bone tissue, partially phosphorylated OPN is sufficient to bind HA and block its growth [32]. Since this function depends on the ASARM motif in a phosphorylation-dependent manner, it is considered a mineralization inhibitor, which can be cleaved by PHEX to block its function. The PHEX protein (coded by a phosphate-regulating gene with homologies to endopeptidases on an X chromosome gene) is associated with X-linked dominant hypophosphatemic rickets (MIM 307800) when mutated. As such, X-linked hypophosphatemia (XLH) is featured by extracellular matrix accumulation of ASARM motifs and osteomalacia [33].

Mice with OPN deficiency do not develop radiological or histological bone changes but show an increased amount of mineral, higher crystal maturity and development of osteoclasts. Therefore, OPN inhibits mineral formation and crystal growth and probably participates in osteoclast recruitment and function [30,34,35,36]. Mice with FAM20c deficiency have increased OPN expression, whose phosphorylation is also affected. In vitro studies show that FAM20c-deficient osteoblasts have OPN down-regulation [17] and hypophosphorylation, which hampers its secretion and, consequently, its extracellular activity [37]. Besides these OPN modifications, other SIBLING proteins are affected, finally leading to a decreased mineralization capacity and reduced cell migration as independent events of systemic phosphorus homeostasis [17].

OPN deficiency also causes parathyroid hormone (PTH) elevation with secondary effects on whole-bone mineral density and cortical bone mass enhancement. Since these features are present in RS patients, they could be related to OPN hypophosphorylation with a secondary inability to block HA formation and growth, as well as PTH induction [38]. Thus, these are potential mechanisms present in RS. On the other hand, OPN participates in hematopoiesis, angiogenesis, proliferation, migration, adhesion and differentiation, as well as in pathologic processes, such as inflammation, cardiovascular diseases, liver fibrosis, cancer, diabetes, wound healing and renal stone formation. When it is produced by immune cells it functions as a cytokine [35]. Although these disturbances have not been described in RS patients, it is not known whether OPN hypophosphorylation due to FAM20C deficiency could affect this non-mineralization process.

### 3.2. DMP1

DMP1 is a secreted acidic phosphoprotein with multiple S-x-E/pS motifs, highly expressed by osteocytes and to a lesser degree by osteoblasts, with roles in osteocyte maturation and bone mineralization through the regulation of osteoblast maturation and phosphate homeostasis, including HA formation in dentine and bone [39,40]. The initially secreted product is an inactive proenzyme, activated upon cleavage at N- and C-terminals, besides other modifications, such as glycosylation and phosphorylation [41]. The PHEX protein is proposed as the enzyme responsible for DMP1 catalytic cleavage [42]. The highly phosphorylated active C-terminal portion mediates calcium recruitment and functions in osteocyte formation and phosphate homeostasis [43]. C-terminal pathogenic variants result in autosomal recessive hypophosphatemic rickets (MIM 241520), featured by rickets, including increased bone density, osteomalacia, short stature, early fusion of cranial sutures, nerve deafness, facial and dental abnormalities, as well as learning disabilities. It is associated with hypophosphatemia, elevated FGF23 and abnormal levels of 1,25(OH)_2_D_3_ [44].

Mice with DMP1 deficiency develop rickets and osteomalacia, including growth plate defects, abnormalities in osteocyte differentiation and the osteocytic canaliculi–lacunae system, an increase of FGF23, mild hypercalcemia with normocalciuria, phosphaturia and severe hypophosphatemia. In bone, calcium and phosphorus are abnormally distributed, with spherical structures such as calculospherulites and with markedly reduced propagation into the surrounding osteoid [17]. It should be highlighted that a high-phosphate supplement diet rescues rickets but not osteomalacia, similar to Hyp mice (the murine model of XLH that lacks the endopeptidase PHEX) and human vitamin D–resistant rickets. Due to this, it appears that DMP1 could negatively regulate FGF23 during bone development and that secondary hypophosphatemia in FGF23 elevation could be the cause of rickets, but osteomalacia depends on other DMP1 functions [42,45,46]. On the other hand, C-terminal DMP1 rescues the null DMP1 mice phenotype. Hence, likely this fragment contains the major biological functions for osteocyte maturation and maintenance of phosphate homeostasis.

DMP1 regulation is through FAM20C phosphorylation (it has 41 phosphates and is highly phosphorylated) [25] and by extracellular phosphate [43,47]. In rats, FAM20C co-localizes with DMP1 C-terminal in Golgi apparatus in young osteocytes, osteoblasts and osteoid. Since the phosphorylated C-terminal DMP1 is present in the Golgi of young osteocytes and localized in canalicular walls in mineralized bone, it could be phosphorylated within osteocytes and secreted into the pericanalicular matrix of mineralized bone, where it recruits calcium ions for mineralization [48]. In addition, mice and osteoblasts with FAM20c deficiency show a decreased expression of DMP1 [28,46]. A non-phosphorylated DMP1 could produce a non-functional protein or undergo a faster degradation, leading to hypophosphatemia, calcium binding alterations and increased PTH. These features related to DMP1 deficiency are similar to those in RS, thus contributing to its phenotype [17,45,49]. However, mice with high serum levels of FGF23, hypophosphatemia, bone and dentin defects due to FAM20C deficiency are not rescued with DMP1 over-expression [46]. Since some SIBLING members are functionally or structurally redundant, there may be function compensations, contributing in a lesser degree to the RS phenotype. Therefore, the DMP1 role should be considered important in RS.

### 3.3. MEPE

MEPE is highly expressed in osteoblasts, osteocytes and odontoblasts, involved in bone and teeth mineralization. It possesses an ASARM motif in the C-terminus (also called Dentonin), a putative signal peptide, an RGD cell attachment motif and a glycosaminoglycan attachment site, as well as several phosphorylation motifs (S-X-E-pS). Full MEPE has many serine residues, an ASARM motif and a polyserine stretch. MEPE has multiple effects, at least in vitro, including an anti-osteogenic effect, bone resorption inhibition, the promotion of phosphaturia modulating serum phosphate, and renal calcification suppression in addition to bone regeneration [50,51]. In vitro, MEPE acts on the inhibition of thes osteoclast formation and phosphate uptake by the kidney and intestine [52,53]. Due to this, it is considered a negative regulator of osteoblasts, bone mass and bone formation [54,55].

The full-length and phosphorylated MEPE promotes HA formation in cell-free in vitro assays; however, it depends on pos-translational modifications as the ASARM motif is derived from MEPE cathepsin B proteolysis and undergoes phosphorylation. Its phosphorylation depends on FAM20C because 31 serine residues are potentially phosphorylated (mainly Ser43, Ser222, Ser362 and Ser441when co-expressed in human cells in vitro,) as well as nine serine residues in the ASARM motif [56]. The phosphorylated ASARM motif binds to HA and prevents mineralization growth and bone resorption, contributing to phosphate regulation through renal and intestinal phosphate uptake inhibition through FGF23 elevation and inhibition of PHEX binding and activity. MEPE also participates in odontogenic differentiation [53,57,58,59]. ASARM–MEPE regulation is achieved by PHEX binding to full-length MEPE or to the ASARM motif-free, preventing its degradation by cathepsin B and ASARM release in the former, while the latter neutralizes its activity through hydrolysis [57,59,60,61]. Therefore, in XLH caused by a PHEX mutation, MEPE is upregulated and ASARM peptides increase and accumulate, causing mineralization defects in bone and teeth [62].

MEPE-deficient mice bear craniofacial abnormalities, that become more evident with age. with a major bone production and trabecular density due to a higher number and activity of osteoblasts, increased bone mass, a smaller and hypomineralized skull, increased density of long bones and grossly visible incisors truncated on radiographs [63]. In addition, these mice are hyperphosphatemic and hyperphosphaturic with reduced FGF23 with age [50,53,64]. On the other hand, MEPE proteolysis produces an osteoregulin domain with the RGD motif for cell attachment, which plays a role in bone homeostasis, probably as a decoy receptor for the preosteoclasts, preventing their differentiation into mature osteoclasts, affecting bone resorption and osteogenesis. The RGD motif also appears to enhance the proliferation of dental pulp stem cells [65].

Due to these different functions, MEPE pathogenic variants are associated with different phenotypes. RGD domain variants causing its degradation or lower expression are associated with hereditary congenital facial paresis, a disorder with uni- or bilateral VII cranial nerve maldevelopment, probably due to a nerve disruption for the diploic thickening of the bony facial canal. Alternatively, variants resulting in RGD and ASARM-truncated protein are associated with adult otosclerosis (MIM 166800), featured by hypoacusia caused by abnormal bone remodeling of the otic capsule [66]. To date, these phenotypes have not been reported in RS, suggesting that hypophosphorylation due to FAM20C deficiency could be partially compensated by other phosphorylation enzymes or functional pathways.

Although FAM20C deficiency does not affect the expression of MEPE by osteoblasts, it could affect its phosphorylation and, therefore, its functions. In such a manner, MEPE hypofunction could contribute to RS phenotype-like osteosclerosis due to higher bone density, higher mineralization and trabecular alterations. On the other hand, FAM20C deficiency has opposing effects on FGF23 and phosphate (hyperphosphatemia and low FGF23) and could counteract hypophosphatemia and FGF23 elevation in RS due to its variability in patients [67,68].

### 3.4. BSP

BSP is a small sulfated and phosphorylated integrin-binding glycoprotein that constitutes the second most abundant protein in the extracellular matrix and approximately 8–12% of non-collagen proteins in the bone. It is the only SIBLING member with specific-mineralized tissue expression and is considered the most potent nucleator of HA at early stages of mineralization. BSP is highly expressed in primary bone osteoblasts, and at lower degree in osteocytes, osteoclasts and chondrocytes in the growth plate, as well as by odontoblasts and cementoblasts in teeth [69]. The BSP structure includes a N-terminal portion rich in serine residues (some of which undergo phosphorylation) and a C-terminal portion with an integrin-binding tripeptide (RGD) sequence that facilitates adhesion of several cell types [70]. The former has a collagen-binding segment and a large number of glutamate residues capable of binding HA to inhibit its growth. Ser136 is a relevant site for its function in mineralization through calcium binding and HA capture by collagen fibers and cells [70,71]. Moreover, BSP promotes osteoblast differentiation [72].

BSP deficiency in mice causes a delay in membranous primary ossification and decreases bone formation and mineralization secondary to defects in osteoblast and osteoclast formation and activity [73,74]. These mice also have lower weight and height, smaller cranium, long bones, wide cranial sutures, delayed skeletal mineralization, thinner cortical bone, decreased growth plate thickness, as well as trabecular bone mass accumulation with age despite lower bone remodeling [75,76,77]. Furthermore, BSP deficiency in mice also results in the delayed repair, mineralization and remodeling of primary bone (slower bone turnover) [78,79]. Although BSP is expressed together with OPN, mice with a BSP deficiency show OPN and MEPE elevation [74]. Regarding mice development, BSP deficiency results in mineralization and endochondral bone alterations due to dispersed and lower mineralization, as well as growth plate defects; as such, these features are probably associated with BSP but not to OPN and MEPE elevation.

On the other hand, FAM20C-deficient mice exhibit increased BSP in cortical bone, which is hypophosphorylated, as shown in in vitro studies in FAM20C-deficient osteoblasts [12,17,28]. Since wide sutures and smaller craniums in mice with BSP deficiency are shared features with RS, BSP hypophosphorylation due to FAM20C deficiency could be a main phenotype contributor.

## 4. FAM20C in Kidney and Parathyroid Gland

### 4.1. FGF23 and 1,25(OH)_2_D_3_

FGF23 is a fibroblast growth factor produced in bone by osteoblasts and osteocytes. It acts on FGF receptors (FGFR1-4), primarily FGFR1c in target cells, with the participation of co-receptor α-Klotho (KL). It is a regulator of calcium and phosphate levels and is produced in the presence of high levels of serum phosphate and 1,25(OH)_2_D_3_. Its activity depends on O-glycosylation at Thr178 by N-acetylgalactosaminyltransferase 3 (GalNT3), which confers protection to furin proteolysis between Arg179 and Ser180 [67]. Its inactivation depends on Ser180 phosphorylation by FAM20C, a modification that prevents O-glycosylation of Thr178 and results in subsequent proteolytic cleavage by furin in circulation [80]. Once released into the bloodstream, at the renal level, the active FGF23 downregulates the type II sodium phosphate cotransporters NPT2a and NPT2c located in the apical membrane of the proximal tubules, in order to decrease phosphate reabsorption [58]. Additionally, FGF23 inhibits the synthesis and favors the degradation of active 1,25(OH)_2_D_3_ through the suppression of 1-hydroxylase and 24-hydroxylase induction, reducing intestinal calcium and phosphorus absorption, respectively. Therefore, the increase in FGF23 produces phosphate wasting and inappropriately low-circulating 1,25(OH)_2_D_3_ levels, including secondary hypophosphatemia and hypocalcemia, ectopic calcifications and impaired bone mineralization.

The gain of function (GoF) mutation of the cleavage site of FGF23 (R176Q/W and R179Q/W) produces FGF23 elevation and is the cause of autosomal dominant hypophosphatemic rickets (ADHR) (MIM 193100), featured by vitamin D-resistant rickets, hypophosphatemia, renal phosphate wasting with low or normal levels of 1,25(OH)_2_D_3_, high alkaline phosphatase (ALP) and osteomalacia. The phenotype has a wide clinical variability, from non-evident alterations to a severe delay in body growth, rickets and deformities, recurrent bone fractures of the lower extremities, osteomalacia, cupping of metaphyseal regions in children and pseudofractures in adults. The age of presentation and FGF23 concentrations can fluctuate between childhood and adulthood, and incomplete penetrance is present too [81,82].

FGF23 is elevated in other diseases, such as XLH rickets (MIM 307800), caused by PHEX mutation; autosomal recessive hypophosphatemic rickets 1 (ARHR1) (MIM 141520) caused by DMP1 mutations; fibrous dysplasia/McCune–Albright syndrome (GNAS1) and cutaneous skeletal hypophosphatemia syndrome (*HRAS, KRAS, NRAS*) [83]. All these entities are characterized by mineralization defects and hypophosphatemia.

### 4.2. PTH

Besides its actions mentioned above, FGF23 also decreases parathyroid hormone (PTH) synthesis and secretion, both of which act together on phosphate regulation. PTH acts on bone, kidney and intestine, and is produced in response to hypocalcemia and hyperphosphatemia, as well as low 1,25(OH)_2_D_3_ and FGF23 [80,83]. In the kidney, PTH acts, similar to FGF23, on proximal tubular cells, through interaction with co-receptor α-Klotho (KL), but it antagonizes FGF23, decreasing the reabsorption of phosphates, increasing 1,25(OH)_2_D_3_ and promoting calcium absorption. In the intestine, PTH stimulates vitamin D-dependent phosphate absorption. In bone, it induces the differentiation and activation of osteoclasts that increase resorption and secondary phosphate liberation [80,83]. However, it is not clear whether FGF23 increases or inhibits PTH secretion [84,85]. In vitro and in vivo, PTH induces FGF23 expression via a Gsα/cAMP–mediated mechanism [86]. Indeed, PTH administration to mice increases 1,25(OH)_2_D_3_, phosphate levels and early FGF23 elevation, but it might cause late and prolonged FGF23 decrease [87]. Hence, FGF23 elevation is caused by high levels of PTH or 1,25(OH)_2_D_3_, as well as hyperphosphatemia and hypercalcemia, but the mechanisms of molecular regulation and time effects are not clear.

In agreement with this, mice with FGF23 overexpression are hypophosphatemic with decreased renal tubular reabsorption of phosphate, increased serum ALP activity, low serum levels of 1,25(OH)_2_D_3_, and low calcium and PTH [88]. However, some studies have found high or low-normal PTH serum levels [89,90,91]. With age, serum and urinary calcium levels diminish slightly and serum PTH and ALP activity increases, without affecting 1,25(OH)_2_D_3_. The bones of these mice show a rickets-like phenotype, including a smaller size, hypomineralization, wider and unmineralized growth plates (as in rickets), severe osteomalacia, increased but hypomineralized trabecular volume, higher cortical bone volume due to unmineralized osteoid and diminished bone resorption with low osteoclast numbers, as well as lower NPT2 at renal proximal tubule epithelial cells. On the other hand, individuals with ADHR have normal PTH with a trend toward high levels, which could be explained by the low 25-hydroxyvitamin exacerbating hypophosphatemia and PTH production [92].

In contrast, FGF23-deficient mice show high serum phosphate and increased renal phosphate reabsorption, elevated serum 1,25(OH)_2_D_3_ due to higher expression of renal 25-hydroxyvitamin D-1α-hydroxylase and increased serum ALP activity, while PTH decreases with age. These mice have a short lifespan, severe growth retardation and abnormal bone phenotype, with hypomineralization, shorter size, markedly wider bone diameter with relatively large epiphysis, osteoid accumulation and secondary thicker trabecular bone [88]. FGF23 deficiency also causes hypoacusia, hypoglycemia, infertility, hippocampal-dependent cognitive function impairment, as well as defects in the thymus and spleen [88,93]. As the whole phenotype cannot be explained by hypophosphatemia, it appears that FGF23 is essential for other functions besides normal phosphate and vitamin D metabolism. Further, FGF23 deficiency in mice mediate OPN increases, contributing to defects of mineralization and bone metabolism [94,95,96].

In the absence of FAM20C, FGF23 is not phosphorylated at Ser180, allowing O-glycosylation at Thr178, which prevents FGF23 degradation and an increase of active plasma FGF23 concentration [80]. In addition to this, FGF23 transcription also increases, as described in osteoblast analysis in vitro [44,97,98]. A higher FGF23 due to hypophosphorylation resembles the GoF mutation, which can translate into hypophosphatemia, hypocalcemia, hyperphosphaturia, low 1,25(OH)_2_D_3_ and secondary osteomalacia/hypophosphatemic rickets.

In RS, hypophosphatemia and hyperphosphaturia, high PTH and 1,25(OH)_2_D_3_ levels occasionally reported could be associated with FGF23 elevation. Although only a few patients have been assessed, its absence in most patients could be explained by variable expressivity factors also acting on ADRH. These differences could be partially attributed to the antagonistic role of PTH, as well to the different mechanisms involved in calcium and phosphate metabolism. Finally, SIBLING proteins, such as DMP1 and MEPE, are antagonistis of FGF23, as DMP1 and MEPE deficiency enhance or diminish FGF23 levels, respectively. All these events point to several dual effects of FGF23 elevation on phosphate and vitamin D metabolism, and partially explain FGF23 effects in some RS individuals [82].

## 5. FAM20C Biological Functions in Non-Mineralized Tissues

Despite the fact that RS defects are due to the above described FAM20C bone targets that act mostly on mineralization, RS patients show other features in non-mineralized tissues. FAM20C participates in multiple organs and in different processes, including wound healing, lipid homeostasis, endopeptidase inhibitory activity, adhesion and cell migration [13], additionally having a potential role in cancer (Table 1) [26,99]. These new targets with different functions in different tissues have been described in recent years, and have not been associated with RS, but they could participate in unexplained RS phenotypes, such as structural brain defects, cognitive impairment or pancreatic and urinary defects. However, features different to mineralization alterations in RS support other FAM20C roles.

### 5.1. FAM20C in Neural Tissue: Neuropeptides

A possible FAM20C activity on neuropeptides of the secretory pathway has been reported in the nervous and endocrine systems. A great number of neuropeptides and pro-hormone precursors undergo phosphorylation as an important modification to modulate their activity [108,109]. These span a high number of peptides at dense core secretory vesicles in the lumen of Golgi that have FAM20C S-X-E/pS phosphosite motifs, which are phosphorylated at various degrees, and include prohormone-derived phosphopeptides. Many of these have not been identified or characterized yet and have unknown functions [110]. Furthermore, FAM20C is responsible for the phosphorylation of the majority of peptides residing within the central nervous system [71,109,111]. It is not known whether FAM20C deficiency provokes their hypophosphorylation, affecting their function, with a possible role in RS.

### 5.2. FAM20C in Neural and Cardiovascular Tissues: Sortilin

Sortilin 1 is another remarkable and interesting target of FAM20C because of its expression in different tissues, its multifunctionality and its role in the pathogenesis of several diseases. Sortilin 1, also called neurotensin receptor 3, is a type 1 transmembrane glycoprotein receptor and a member of the vacuolar protein sorting 10 protein family of receptors, encoded by the *SORT1* gene. It is ubiquitously expressed, particularly in the liver, adipocytes, vascular tissues, immune system, heart and skeletal muscle, and has a high and broad brain expression throughout development [29,112]. In the adult brain, it is present in cerebrum and subcortical structures, with differential regional and laminar patterns, besides microglia, as well as in accordance to neuronal size, types and centers of descending/ascending neuroanatomical pathways. These expression differences by the cell and structure lead to the assumption that it could function in different processes accordingly.

Sortilin 1 structure consists of a N-terminal Vps10p domain, a single transmembrane domain and a shorter C-terminal cytoplasmic tail. It contains a S-X-E/pS site (S825) in the C-terminal intracellular domain tail, which is a phosphorylation site for FAM20C and casein kinase 2 (CK2) [113]. Its primary role is as a sorting receptor that regulates intracellular protein transport in the Golgi apparatus, lysosomes and endosomes. It plays roles in cell survival and signal transduction. Sortilin 1 has been associated with vascular calcifications, cardiometabolic diseases such as lipoprotein metabolism dysregulation, coronary artery disease, atherosclerosis, obesity, type II diabetes, neurological diseases such as Alzheimer-type dementia, Parkinson’s disease, depression, oncologic diseases, as well as immune processes. Its role in hypertension, dyslipidemia and diabetes is well established, as patients with cardiovascular risk factors have high circulating sortilin levels [29].

Sortilin 1 Ser825 phosphorylation was identified as a CK2 [113] and years later as a FAM20C target, with a role in vascular calcification [106]. Sortilin 1 overexpression was found in human calcified atheroma. Smooth muscle cell assays in vitro demonstrated sortilin 1 in extracellular vesicles of microcalcification areas, correlating with calcification and tissue nonspecific alkaline phosphatase (TNAP) activity. This effect was independent of cholesterol levels and immune cell sortilin 1 production. Highly phosphorylated sortilin 1 directly correlates with the calcification and TNAP activity, and its phosphoserine state depends on FAM20C. Human calcified carotids and human smooth cells overexpressing FAM20C have increased pSer825. On the other hand, FAM20C inactivation does not affect TNAP activity, probably as a compensatory mechanism mediated by CK2 kinase or additional kinases [72,114].

This is supported by the GoF Ser825 mutation resulting in higher TNAP activity, and the LoF Ser825 mutation resulting in the absence of TNAP activity alteration or calcification enhancement. Since the phospho-null variant at this site yields a sortilin 1 trafficked to the endosomal system for degradation, it is possible that sortilin 1 phosphorylation controls its trafficking and subsequent TNAP transportation into extracellular vesicles for enhancing mineralization. The whole vascular calcification includes other molecules, since the process implies the trans Golgi network traffic regulation by phosphorylated sortilin 1 in order to promote TNAP transportation to specialized membrane domains containing caveolin-1, by means of Rab11-enriched endosomes. Finally, the sortilin 1/TNAP complex is found in extracellular vesicles [106].

Due to these broad functions, we believe that sortilin 1 could play a role in the vascular and brain tissue abnormalities of RS. However, the phosphorylation state of Ser825 due to FAM20C deficiency in RS is unknown, and so is if a possible rescue of Ser825 levels by casein-kinase 2 leading to normal sortilin 1 activity. Finally, soft tissue calcifications in RS are predominantly found in the brain, including intraparenchymal and perivascular regions, and are rarely found in blood vessel walls [8,9,115]. On the other hand, as higher sortilin 1 expression is associated with vascular calcifications, sortilin LoF could protect or reduce calcification, thus soft tissue calcifications in RS may be sortilin-independent.

Arterial calcification is also described in OPN deficiency or hypophosphorylation [116]. However, tumor microcalcifications in the form of hydroxyapatite have also been associated with high OPN expression, without a known mechanism [117,118]. These contradictory actions stress the need to study sortilin 1 and OPN phosphorylation states in soft tissues, including the brain, which has high expression of OPN and sortilin 1, to elucidate the ectopic calcifications observed in RS. Indeed, sortilin 1 Ser825 hypophosphorylation could have a protective effect on vascular calcifications, a phenotype that we believe should be intentionally evaluated in patients with non-lethal RS as they age [17,96]. Moreover, it is important to highlight that mice with sortilin 1 deficiency do not show cortical or trabecular bone alterations, and osteoblast and osteoclast functions are not affected. The association of LoF sortilin with a reduced vascular calcification and no bone metabolism effects, suggests a protective role in vascular mineralization and that RS vascular wall calcification could depend on other pathways.

As previously mentioned, sortilin 1 is a multifunctional protein with high and broad functions in neural tissues. As a VPS10P-domain receptor family member, sortilin 1 has roles in neuronal functions and viability mediated by different mechanisms, including the response to a broad range of ligands, such as trophic factors and neuropeptides, besides transmembrane proteins [119]. Neurotrophins trafficked by sortilin 1 to the plasma membrane, lysosomes or endocytic pathways, contribute to the development, function and plasticity of the nervous system through interactions that include cell death signals and axon anterograde transport, among others. It plays a role in Alzheimer’s dementia though β-secretase trafficking leading to an increased cleavage of β-amyloid precursor protein. This process is mediated by the regulation of the β-secretase-amyloid precursor protein-cleaving enzyme 1 (BACE1), one of the enzymes that cleaves the β-amyloid precursor protein (APP). This is supported by a positive correlation between increased cleavage of APP and high sortilin 1 levels [120], as well as extracellular deposits of the sortilin 1 C-terminal in human brains with cerebral amyloid pathology [121].

### 5.3. FAM20C in Brain: Sortilin 1 and Sonic Hedgehog (SHH)

An interesting protein affected by sortilin 1 is SHH, a ligand of a pathway with roles in fetal development and patterning, with prominent roles in neural tissue. Sortilin 1 interacts with SHH in the Golgi, where it promotes its accumulation and reduces its transport through the secretory pathway. In fact, the midline phenotype associated with SHH deficiency in mice is rescued by sortilin 1 deficiency. Thus, sortilin 1 acts as a negative regulator of SHH secretion in vivo. Reduced SHH secretion also occurs in axons and SV2+ vesicle pathways, independently of trafficking to lysosomes or endosomes and some axon trafficking pathways (regulated by BDNF). Thus, sortilin 1 deficiency could increase SHH secretion, favoring this pathway [122].

In mice, selective FAM20C deficiency in the brain produces a phenotype similar to RS, with intracerebral calcifications, accompanied by microgliosis and astrogliosis, events independent of hypophosphatemia [123]. If brain sortilin 1 hypophosphorylation is present in RS patients, it could result in SHH dysregulation, with effects on the forebrain development. These effects could be associated with some of the RS structural brain defects found in some patients. However, as the frequency of brain defects in RS is low, they could depend on the genetic background of each patient. A hypothesis to consider is that RS brain defects associated with SHH disturbances could be a pathogenesis starting point. Given the relevance of sortilin 1 functions in the nervous system and considering the presence of seizures and intellectual disability in some patients with RS, another hypothesis to consider is a possible effect on sortilin 1 phosphorylation in FAM20C deficiency.

### 5.4. FAM20C in Cardiac Tissue: HRC, STIM1 and CSQ2

Many sarcoplasmic reticulum (SR) proteins contain S-x-E motifs, which are potential FAM20C substrates. The histidine-rich calcium-binding protein (HRC) was the first SR protein involved with FAM20C. HRC is a luminal protein of the SR, specifically phosphorylated by FAM20C in Ser96 of an S-X-E/pS motif, which is expressed in striated and arteriolar smooth muscles [107]. HRC participates in the entry and exit of calcium in the SR in the myocardium through the recognition of sarco/endoplasmic reticulum Ca^2+^ adenosine triphosphatase-2a (SERCA2a), a Ca^2+^ ATPase pump responsible for the uptake of Ca^2+^ by the SR, through a mechanism dependent on phosphorylated Ser96 and on ryanodine receptor 2 through triadin, which are also regulatory proteins of SERCA2a. HRC phosphorylation is very important, since the Ser96Ala variant disrupts phosphorylation and is associated with ventricular arrhythmias in patients with dilated cardiomyopathy and deaths due to arrhythmias, with a greater frequency in homozygous than heterozygous states [124,125]. In addition, HRC is related with autosomal dominant isolated cardiac conduction [126] and the familial heart block type 1 [124,127].

Other proteins involved in Ca^2+^ signaling are calsequestrin 2 (CSQ2) and stromal interaction molecule 1 (STIM1), both regulated by FAM20C; they are also ER proteins with Ca^2+^ signaling functions in cardiomyocytes and other cell types. CSQ2 is considered the major Ca^2+^ binding protein in the SR and contains the S-X-E/pS (Ser385) motif on its C-terminal tail, which is a region for calcium binding and polymerization. STIM1 is a protein located in the lumen of the SR, controlling cellular Ca^2+^ content through the regulation of store-operated Ca^2+^ entry and other sensing mechanisms, and has a serine at 88 position. FAM20C phosphorylates CSQ2-Ser385 and Stim1-Ser88 to induce their activity. Moreover, FAM20C deficiency in cardiac tissue impairs the phosphorylation of both proteins, leading to heart failure induced by age and pressure overload [107].

It is not known whether the phosphorylation levels of HRC, CSQ2 and STIM1 are affected in RS patients, among other proteins that could be targets of phosphorylation and that participate in the regulation of calcium at the SR level in the heart. We consider that HRC deserves more attention due to the risk of lethal arrhythmias which could be associated with early lethality in RS, mainly in those FAM20C variants that produce mislocalization or LoF kinase activity [124].

### 5.5. FAM20C in Metabolism: PCSK7 and PCSK9

FAM20C transcript is overexpressed in carotid atherosclerosis symptomatic plaques, as well as in proprotein convertase PCSK6, in correlation with genes associated with inflammation and matrix degradation during plaque rupture [128]. PCSK6 belongs to the secretory basic amino acid-specific subtilisin–kexin-like proprotein convertases (PCs or PCSKs), constituted by seven members, including PCSK7 and PCSK9, which are FAM20C targets.

PCSK7 is ubiquitously expressed, with actions on many different proteins involved in different pathways and in development [129]. It is active at the cell membrane and Golgi levels and is associated with plasma triglyceride level regulation. GoF variants are related to higher expression, in turn associated with high levels of the protein, while LoF variants are related to lower triglyceride levels [130]. One of the triglyceride regulation mechanisms is mediated by PCSK9 binding to apolipoprotein A-V (ApoA-V) to favor its lysosomal degradation and to reduce its secretion. ApoA-V stimulates lipoprotein lipase activity, to activate TG clearance. FAM20C phosphorylates PCSK7 at Ser505 when the LoF variant Arg504His is present, resulting in a lower degradation of apoA-V in the liver, with a secondary higher ApoA-V secretion and circulation, higher lipoprotein lipase activity, increased TG content in adipocytes and lower triglyceride levels [105]. PCSK9 is highly expressed in liver, with participation in cholesterol metabolism and cardiovascular risk. PCSK9 activity is regulated by FAM20C phosphorylation, which acts on serines 47, 666, 668 and 688 [104]. In the ER, its N-terminal prodomain undergoes autocatalytical cleavage, after which it is secreted with the mature catalytic domain as an inactive protease. The latter is secreted as a complex with the mature catalytic domain, keeping it as an inactive protease. Secreted PCSK9 is re-uptaken by the hepatocyte, entering and sorting the cell membrane LDL receptor into endosomes and lysosomes for degradation [131]. The heterozygous GoF mutation favors a higher LDL receptor binding, uptake and degradation in endosomes or lysosomes, resulting in hypercholesterolemia with high LDL cholesterol [132]. On the other hand, PCSK9 LoF variants impair the LDL receptor’s sorting and degradation, resulting in a higher cholesterol clearance and subsequent lower circulant LDL levels [133]. The phosphorylation outcomes are similar to LoF, decreasing LDL receptor degradation followed by increased secretion and hepatocyte activity, favoring LDL cholesterol clearance and hypocholesterolemia [104].

It is not known whether RS patients have changes in PCSK7/9 phosphorylation and circulant triglyceride/cholesterol levels. PCSK9 hypophosphorylation could represent a LoF variant, and RS patients could have reduced LDLR degradation and low LDL cholesterol. It is possible that PCSK7 has an effect only in the presence of the R504H variant.

### 5.6. FAM20C in Endoplasmic Reticulum–Redox Homeostasis: ERO1a

Ero1a (oxidoreductin 1 alpha) is a sulfhydryl oxidase protein of the ER that catalyzes the formation of disulfide bonds that plays a role in oxidative folding of proteins. An initial study in vitro in HeLa and HepG2 cells linked secretory protein phosphorylation with oxidative protein folding. Ero1a is phosphorylated by FAM20C at Ser145 in the Golgi within the C-terminal S-x-E/pS recognition motif (in the outer active site-containing flexible loop). This phosphorylation confers greater oxidation activity to Ero1a and promotes oxidative protein folding. Ero1a deficiency alters the redox regulation in the ER, and FAM20C deficiency affects the ER–redox homeostasis. In addition, FAM20C is known to interact with up to 349 ER and Golgi proteins with roles in protein processing, N-glycan biosynthesis and fatty acid metabolism [49]. Although no Ero1a changes have been examined in RS, these data further extend the possible effects caused by FAM20C deficiency and open the door for the detection of new FAM20C targets.

### 5.7. FAM20C in Clotting: vWF

Von Willebrand factor (VWF) participates in the maintenance of hemostasis during vascular damage and favors platelet adhesion to the endothelium. Defects in this factor are related to hemorrhagic diseases, such as Von Willebrand disease, thrombotic thrombocytopenic purpura and HELLP syndrome (hemolysis, elevated liver enzymes and low platelet). VWF contains three domains necessary for platelet binding, with domain A2 having two FAM20C phosphorylation sites (S1517 and S1613) at the S-X-E/pS recognition motif in vitro. S1613 is the major phosphorylation site and enhances stable platelet adhesion [103]. No coagulation abnormalities have been reported in RS, which could be explained by the possible action of other compensatory kinases that act in vivo at the same site as FAM20C. It would be of interest to examine whether FAM20C deficiency has any impact on VWF, although, at present, there is no clinical evidence [103].

### 5.8. FAM20C in Salivary Glands: BMP4

FAM20C is expressed in the cytoplasm of epithelial ductal cells and has also been described in the development and function of mouse salivary glands. FAM20C phosphorylates salivary acidic proline-rich phosphoprotein-1 (PRP1), a secreted protein contained in saliva, to bone morphogenic protein-4 (BMP4), a member of the transforming growth factor–β superfamily. Deficiency of FAM20c in salivary gland cells produces developmental and functional defects, including both structural and morphological changes, such as smaller lobules, increased number of ducts with prominent structure and increased mesenchymal tissue in newborn mice; and, at 8 weeks, increased mesenchymal tissue and reduced acinar cells. It also affects the maturation of granular convoluted tubule cells and the accumulation of secretory granules, with aberrant morphology and a reduced size. Functionally, it causes duct dysfunctions manifested by a reduced salivary flow rate and elevation of the Na^+^, Cl^−^ and K^+^ concentrations. In addition, it affects the expression of BMP2 and BMP7 proteins (BMP signaling pathways) [134]. These results link FAM20C to other biological roles, as well as a possible role in salivary glands in RS. Possible targets and features need to be studied.

## 6. FAM20C Pathogenic Variants

In 2007, pathogenic variants in *FAM20C* (NM_020223.3) were identified as the cause of RS (OMIM # 259775) [135], secondary to the presence of a chromosomal rearrangement with homozygous 7pter deletion in one case. Since FAM20C was the candidate gene responsible that was contained in the deleted region, variants in exonic regions were analyzed and validated in other patients [1]. Although it was initially described as a lethal syndrome, FAM20C variants in children with subtle RS phenotype were reported in 2009. Thus, two types of RS are recognized according to survival: lethal (LRS) and non-lethal (NLRS). Since then, different pathogenic variants have been detected for both types [136].

**Figure 3:**
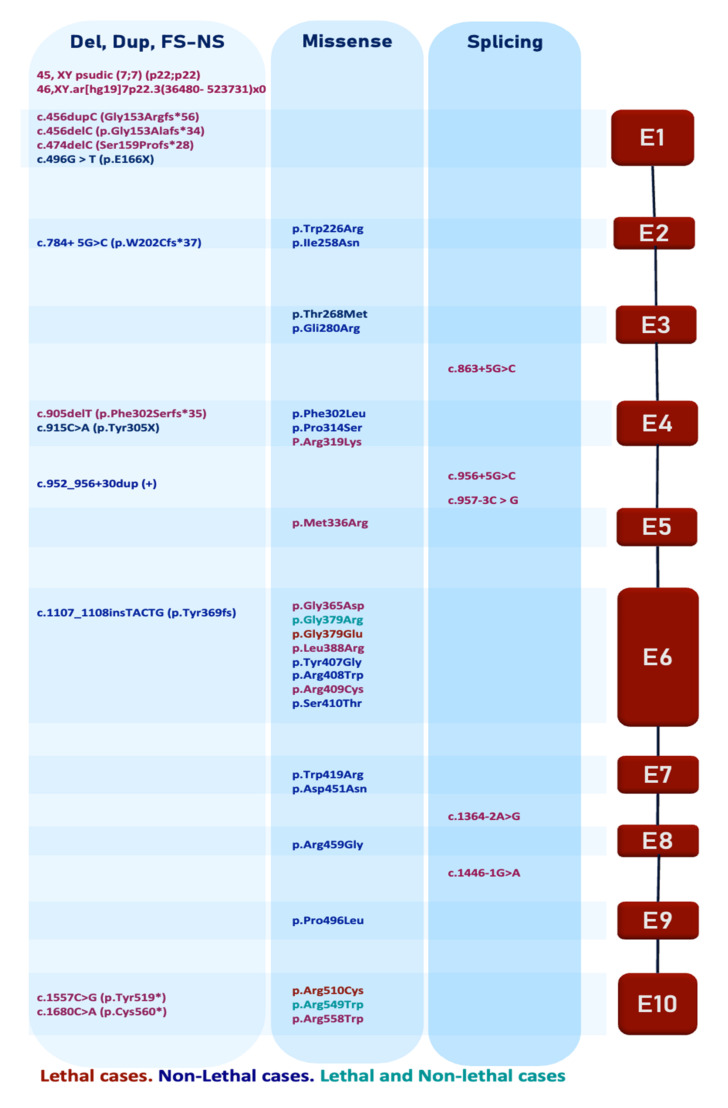
Sequence localization and type of FAM20C pathogenic variants described in Raine syndrome cases.

To our knowledge, a total of 42 different variants have been described to date, including lethal and non-lethal cases (listed in Table 2 and Figure 3). These include different types of variants which correspond to missense (24), splicing defects (5), truncated proteins by indels out of frame (8), non-sense (4) and chromosomal rearrangements (2). All exons harbor pathogenic variants, exon 6 (181nt sequence) being the richest with nine variants, including four in lethal and four in non-lethal cases. Shorter exons harbor only one pathogenic variant (exon 5 (116nt), exon 8 (82nt) and exon 9 (60nt)). In addition, two missense variants are present in NLRS and LRS.

From a total of 22 exclusive lethal variants, absence, nonsense or truncated types are the most frequent (10). Variants producing short transcripts potentially undergo non-sense-mediated decay, resulting in absent or truncated proteins (gene deletion and indels out of frame) with secondary KD absence. These FAM20C variants include two homozygous gene deletions [1] (45, XY psudic (7;7) (p22;p22) and a 487-kb homozygous deletion in 7p22 [137] in two cases, and one compound heterozygous case with variants predicted to produce very short proteins of 206 and 186 aa (c.456dupC/Gly153Argfs*56 and c.474delC/Ser159Profs*28) [139], one case with a homozygous variant c.456delC (p.Gly153Alafs*34) in exon 1 producing a protein of 190aa [137], one case with an homozygous frameshift variant in exon 4 (c.905delT, p.Phe302Serfs*35) producing a 337 aa protein [138] and one case with a homozygous nonsense variant in exon 10 (c.1557C>G, p.Tyr519*) producing a protein of 519 aa, with incomplete KD that keeps 165 aa and lacks 46 aa from the KD 5′end. The variant c.1680C>A p.Cys560* was identified in two unrelated cases [153,154], affecting only the last 5 aa from the KD 5′end. As the KD domain starts at 354 aa and ends at 565 aa, only two of these variants keep an incomplete KD. Thus, lethality is probably associated with the absence or important impairment of kinase activity (due to biallelic non-sense or truncated variants) that are not compensated by missense variants, as in the non-lethal cases, which have non-sense or truncated proteins in a heterozygous state with missense variants that keep the KD.

The missense variant c.1135G>A (p.Gly379Arg) is present in one lethal and one non-lethal case. It was identified in one lethal homozygous case who survived less than 10 days and in one 3 y/o non-lethal homozygous patient with a severe phenotype [136,148]. Therefore, we assume that this variant could be associated with a severe RS phenotype. From seven splicing defects, two are non-lethal. One mild case presents c.952_956+30dup resulting in a mild alteration of splicing by the usage of a predicted second downstream splice donor site, but with a second heterozygous missense variant (c.906C>A, p.Phe302Leu) [5]. The other variant is a homozygous FAM20C donor splice site mutation c.784+5G>C/pW202Cfs*37, reported in four siblings with severe dental defects but mild dysmorphic facies and without evident skeletal abnormalities [141]. The first splicing variant was recognized as a potential polymorphism found in a homozygous state, but another variant was needed for producing the RS phenotype, while the second, despite resulting in a frameshift and premature stop codon prediction, resulted in a partial effect on exon skipping showing expression of a shorter size and the wild-type sized transcript. Thus, they are probably hypomorphic alleles, producing a mild effect.

When considering non-sense or truncated variants in non-lethal cases, there are only three families. One of the families has a non-sense mutation in exon 4, producing a 305 aa protein (c.915C>A, p.Y305X (p.Tyr305X)), but is heterozygous with a missense variant c.803C>T, p.Thr280Met located in the ATP-binding P-loop [143]. The second family presents a variant that produces a 239 aa protein (c.784+5G>C p.W202Cfs*37) [141]. It is necessary to point out that there are non-sense/truncated biallelic cases likely leading to non-viable proteins or that produce proteins with KD absence; thus, the missense variant has likely a less deleterious effect on FAM20C functions and the resulting phenotypes, explained in part by the presence of the second allele producing the protein with KD, in all cases.

Regarding non-lethal and heterozygous cases, a frameshift insertion c.1107_1108insTACTG (p.Tyr369fs) and a missense substitution c.1375C>G (p.Arg459Gly) were the initial reported variants in a middle-aged patient presenting classical but milder symptoms of Raine syndrome [147]. Despite its localization on the conserved C-terminal domain of FAM20C, the resulting mild phenotype points out that these variants have a milder effect on the protein or that additional factors can compensate for the lack of kinase activity, softening the phenotype. Although 16 variants are localized in the KD domain—8 lethal, 7 non-lethal and 1 shared—a potential effect on KD activity is predicted in 67.6% of all the variants, being more prevalent in lethal (76.4%) than non-lethal RS (58.8%). It is suggested that mutations that result in impaired kinase activity are pathogenic and that the extent of activity impairment is related with phenotype severity [3,12].

In vitro assays with some RS variants result in the mislocalization of FAM20C from Golgi to the ER, with undetectable secondary secretion into the media, while other variants remain in Golgi but have low or absent kinase activity. Since misfolded secretory pathway proteins are typically retained in the ER and some are selected for Golgi quality control, variants producing misfolded FAM20C probably remain in the ER or are targeted for lysosomal degradation [155]. From these assays, lethal homozygous variants Gly379Arg/Gly379Glu have no detectable kinase activity, and Lys388Arg/Arg549Trp show mislocalization to the ER and no secretion into the media. On the other hand, the non-lethal heterozygous variants Ile258Asn/Gli280Arg, of one case, are associated with diminished secretion and non-kinase activity for the first, while the second variant has diminished secretion and kinase activity, supporting a possible residual kinase activity in non-lethal cases [11]. Since the homozygous variant Asp451Asn from a non-lethal case results in mislocalization in the ER and non-secretion into the media, similar to lethal variants, a direct relation between kinase activity and degree of secretion to phenotype is ruled out, although the phenotype in this non-lethal case is severe [12]. An interesting variant is reported in a 70 y/o patient with a very mild phenotype with the heterozygous variant c.906G>A (p.Phe302Leu) and a duplication c.952_956+30dup at the end of exon 4 of the FAM20C gene [5]. This second variant is considered a common polymorphism and could produce a novel downstream splice donor site with effects on splicing leading to a premature stop codon, contributing slightly to the phenotype.

Although most missense variants result in lower kinase activity, variants corresponding to lethal cases are more frequently secretion defective [12,27]. However, there is no correlation between the in vitro secretion and kinase activity. These pinpoint that some variants may preserve folding properties but affect kinase activity. Variants affecting its folding and secretion, or the complete absence of kinase activity, could cause a severe phenotype or lethal RS, whereas a mild or non-lethal phenotype could be due to variants producing proteins with preserved folding and secretion properties, but diminished kinase activity. This is supported by the fact that FAM20C was undetectable in both liver and pancreas from one RS patient with biallelic truncated variants (187 and 206 aa proteins) [139].

Considering these variant aspects, there are no data so far to support a genotype–phenotype correlation. Together, these observations suggest that the extent of the impairment of kinase activity correlates with disease severity, but a threshold or a minimal partial protein activity is probably required for a non-lethal phenotype, plus the extent of the misfolding and secretion. Furthermore, as the Gli379Arg homozygous variant is present in one lethal and one non-lethal case, the participation of modifier genes, epigenetic factors or compensative kinases could be in action. To identify the lethal and non-lethal associated variants, it is indispensable to evaluate expression and activity of missense variants in KD for both phenotypes.

## 7. Clinical Aspects of RS

In 1989, Raine described a neonate with lethal cranial anomalies and osteosclerosis, Kingston described another case in 1991 and Kan used the eponym “Raine syndrome” while reporting another case in 1992, which was incorporated into *Online Mendelian Inheritance in Man* (OMIM) formally as osteosclerotic bone dysplasia, lethal (OMIM 259775) [135,156,157,158]. However, in 1985, Whyte reported two infant sisters with a lethal condition called “congenital sclerosing osteomalacia with cerebral calcification” (CSOCC), incorporated one year later as “osteomalacia, sclerosing, with cerebral calcification” (OMIM 259660). Thus, Whyte reported the first case named as CSOCC and Raine’s report was a second case, but it was thereafter commonly named RS. Although initially featured as a lethal disorder, in 2009, new non-lethal cases were described [135]. To date, RS is defined as a disorder with an autosomal recessive inheritance, belonging to the group of hereditary sclerosing bone dysplasias that include osteopoikilosis, osteopetrosis, osteopathia striata, progressive diaphyseal dysplasia, pyknodysostosis and hereditary multiple diaphyseal sclerosis. It is also classified into the neonatal osteosclerotic dysplasias, group 22 of the *nosology and classification of genetic skeletal disorders 2019*, which also include Blomstrand dysplasia, desmosterolosis, Caffey’s disease (including prenatal, infantile and attenuated) and Caffey’s dysplasia (severe variants with prenatal onset) [73].

RS has an estimated prevalence of <1 in 1,000,000 [3], and 70 cases have been reported to date, around 40 LRS and 30 NLRS, a wide clinical heterogeneity that is more evident in NLRS, which include age-related features. In accordance with recessive inheritance, the history of consanguinity is described in ~50% (19/40 LRS and 18/30 NLRS). Although most cases are detected at birth, facial alterations that are Binder-like (such as a flat facial profile, hypoplasic nose and prominent eyes) or Crouzon-like have been described prenatally, in addition to cerebral alterations, such as large choroid plexuses and an echogenic appearance of the brain, indicative of intracerebral calcifications [7,8,9,10,11,138,153].

### 7.1. Lethal Raine Syndrome

LRS is well characterized by generalized osteosclerosis with a periosteal bone formation, characteristic facial dysmorphisms and intracerebral calcifications. Table 3 and Table 4 compile the craniofacial and extra-craniofacial features of RS. Diagnosis is achieved early, in the first hours or days of life. Individuals of Arab origin have the highest number of cases, followed by Caucasians and Latinos.

### 7.2. Craniofacial and Skeletal Features

The craniofacial phenotype is the most striking feature of RS. The most common feature is ocular proptosis (100%) and mid-facial hypoplasia (95%), followed by a depressed nasal bridge (85%), brain calcifications (70%), wide fontanelles and micrognathia (75%), atresia or stenosis of choanae or piriformis (62%). Palate defects are frequent (64%), including a cleft (31%) and a high narrow palate. Lower frequency but still relevant features include microcephaly, a fish-like or tented mouth, gum hyperplasia, a high palate and low set ears. Table 3 summarizes clinical features of the 40 cases. Dental defects are rare in LRS due to perinatal lethality, with reports of only two cases: one 2y/o survival case (with teeth hypomineralization and an enlarged pulpar chamber) and a newborn with dysplastic and loose natal teeth [10]. Skull growth and mineralization defects due to hypophosphorylation of SIBLING proteins is considered the primary abnormality, causing RS characteristic deformities due to the spatial and temporal restriction of brain and facial structures [79].

Among extra-craniofacial features, skeletal alterations are the most common, as enlisted in Table 4. The most frequent are long bones and skull sclerosis (>90%), followed by a periosteal reaction (67%). In addition, a short thorax and neck are frequent, and shorter limbs, vertebral defects, fractures and pseudo-fractures are less frequent. Moreover, there are some uncommon features, such as bone-in-bone images and brachydactyly. Among bone metabolic disturbances, ALP elevation was detected for the first time in 1996, and since then other new cases with hypophosphatemia, low serum calcium and 1,25-dihydroxycholecalciferol and PTH elevation have been identified. However, most reports do not describe them. The sum of hypophosphorylation of SIBLING proteins is the main factor contributing to these features [168,169].

### 7.3. Extra-Skeletal Features

Brain structural defects have been described in 10 cases, including cortical dysplasia, cerebellar hypoplasia, gliosis, ocular defects, corpus callosum and hypophysis dysgenesis (Table 7). These abnormalities cannot yet be explained by SIBLING protein effects on neural tissues, and other pathways could be involved, as described above. Among other extra-skeletal phenotypes, respiratory alterations are very common and important, including a small thorax, lung hypoplasia and choanal atresia or stenosis, which contribute to respiratory failure and death. Uncommon features, include heart defects and pulmonary hypertension, genito-urinary defects (in the kidney and ureter, cryptorchydia and microscrotum) and calcifications in abdominal organs (ovary, kidney, liver and adrenal glands). Many of these features might be subdiagnosed, as they are not described or ruled out due to early mortality. Since SIBLING deficiencies are not associated with extra-osseous defects, other FAM20C targets with roles in heart and genitourinary structures could be involved, as well as other organs, such as the pancreas or thymus (i.e., there is one RS case with an absence of islets of Langerhans), supporting FAM20C actions on spatial and temporal development, which have not been attributed to RS until now [28,44,135].

### 7.4. Survival

The majority survive less than 24 h (40%), 1-7 days (15 %) and 8 to 30 days (15%). The longest survival was seen in two cases of 8 months and 2 years, respectively. The cause of death is respiratory failure (73%), due to the obstruction of the upper airway (choanal and/or pyriform stenosis or atresia), and pulmonary hypoplasia, but this could be higher as some cases did not report the cause of death. Although death in RS is related to respiratory causes, the role of HRC in arrythmias and other cardiovascular FAM20C targets mentioned above could play a potential role for poor survival [124].

Summarizing the clinical and radiological features, there are four major clinical aspects to consider in RS: the craniofacial phenotype, bone sclerosis, periosteal reaction and respiratory disturbances. Among craniofacial abnormalities, the most typical in RS are ocular proptosis and nasal hypoplasia with choanal atresia/stenosis. Finally, broad sutures and bone sclerosis complete the core features of lethal RS. Thus, bone sclerosis and the craniofacial phenotype allow us to suspect and identify severe or lethal RS at birth very easily, ruling out mainly bone disorders, such as Crouzon syndrome, osteopetrosis dysplasia, desmosterolosis and congenital cytomegalovirus infection [10,135]. Due to poor survival, abdominal and brain evaluations are not made in most cases, and neither are bone metabolic screenings. We stress the need to consider them as extra-skeletal features are probably more frequent. Due to early lethality, some cases might go unreported, so the frequency of RS could be higher. We believe that all cases with craniofacial RS-like (mild or severe) and bone sclerosis should include a wider approach to look for brain calcifications and structural defects, soft-tissue calcifications and heart and genitourinary defects, including a complete bone metabolic profile.

## 8. Non-Lethal RS Phenotype

The first two non-lethal cases reported in 2009 correspond to two children, 8 and 11 y/o, from two unrelated families, with features suggestive of RS, including craniofacial RS abnormalities at birth or developed during growth. To date, 30 cases have been described, that show a very wide phenotypic variability that favors a delay in diagnosis, which has been made as early as three months and as late as 72 years [5,6,136]. NLRS-affected individuals are mostly from Arab populations, but also from Latin, Asiatic, African and European countries. Consanguinity is present in 60% of the cases, although in some reports it is not described.

### 8.1. NLRS Craniofacial and Skeletal Features

The most frequent craniofacial features of NLRS include cerebral calcifications (76%), mid-facial hypoplasia (72%), ocular proptosis (66%), a depressed nasal bridge (52%) and abnormal teeth (58%). Choanal atresia/stenosis, a high palate (50%), micrognathia, low set ears, hypoplastic nose and hearing loss (30%). This facial phenotype is reminiscent of LRS, although milder. These features are summarized in Table 5. Dental abnormalities are important due to their frequency and degree, but also their variable expressivity, including permanent teeth with yellow brownish discoloration to spontaneous abscesses and loss of teeth, tooth decay, retention of canines, delay in permanent teeth eruption or unerupted permanent teeth, ectopic eruption of premolars (probably due to a reduced space resulting from micrognathia), incisal notches in the central incisors and vertical ridges and grooves. Deciduous teeth are small and yellow, with incomplete root formation, enlarged pulp chambers and root canals with impaired apex formation, periapical and periodontal abscesses and pulpal calcification. Thin and poorly mineralized, hypoplastic enamel and with pitting and interglobular dentine are features that are shared with amelogenesis imperfecta IC [170].

### 8.2. Extra-Craniofacial Skeletal Features

Among other bone features, osteosclerosis is the most frequent (86%), but has a wide variability since it can be localized or generalized. Other skeletal alterations, such as periostic reaction, vertebral defects and short limbs have frequencies of 10%. Furthermore, uncommon features, such as prognathism, pectus excavatum, a small thorax, osteomalacia and osteonecrosis can be present (listed on Table 6). Mild to severe respiratory problems, although not frequent, are present in 27%. Bone metabolic abnormalities are reported in some cases, such as hypophosphatemia, hypocalcemia, low 25-OH Vitamin D, and elevation of FGF23, PTH and ALP, as well as hyperphosphaturia.

### 8.3. Extra-Craniofacial and Skeletal Features

Among neurological defects, brain calcifications and microcephaly are frequent (76% and 36%, respectively), followed by functional alterations, such as developmental delay or intellectual disability (46%), hearing loss (30%) and seizures (26%), and with a lower frequency of structural brain defects, hydrocephalus and visual defects (Table 7).

The most atypical and milder case corresponds to a 72 y/o man, who had a spontaneous, rapidly progressive osteonecrosis of the knee with no dysmorphic face nor clinical signs of dysplasia. A *FAM20C* mutation was detected after the sequencing of 409 genes related to skeletal disorders, but in a heterozygous state with a missense variant and a polymorphism mentioned above [5]. Another atypical case is a short-statured (<2.8 SD) 61 y/o man with hypophosphatemia, osteomalacia, increased periosteal mineral density and bone formation in the femoral neck and lumbar vertebrae, with a history of mild bowlegs in childhood, all teeth lost by the age of 17, hypophosphatemic hyperphosphaturia incidentally detected at 27 years, ossification of the posterior longitudinal ligament at the age of 35 (associated with alphacalcidol and phosphate treatment) and nephrocalcinosis at 41 years (attributed to phosphate therapy) [83].

Summarizing the major NLRS features, they span from milder facial phenotype, brain calcifications and bone mineralization defects, to intellectual impairment, hearing and visual defects. From 30 NLRS cases, almost 50% have functional defects [4,141,142]. Among mineralization defects, dental and bone anomalies are important for NRLS diagnosis, which also include osteomalacia and hypomineralization, ectopic calcification of pericoronal soft tissues, enamel and dentin defects.

According to the wide phenotypic spectrum, the non-lethal cases could be classified as mild, moderate or severe, with/without intellectual impairment or even as bone-restricted or with dental phenotype dominance. In recent years, more cases have been identified due to genomic diagnosis. As more cases become reported, the phenotype will probably be tuned up with new data. For now, due to this great variability, we consider that patients with amelogenesis, dentinogenesis and mineralization bone defects, with or without learning impairment or RS-like facial features, should be approached as phosphorus defects including Raine syndrome, and must undergo biochemical bone profiles [175].

## 9. Differences between Lethal and Non-Lethal RS

It is clear that survival time is the first difference between LRS and NLRS, which differ also in frequency and severity as well as in the presence/absence of other features. Facial features are milder and less frequent in NLRS, such as ocular proptosis (100% vs. 66%), hypoplastic/short nose (57% vs. 40%), microcephaly (40% vs. 36%), micrognathia (72% vs. 36%) and gum hyperplasia (47% vs. 15%). However, milder and more frequent are choanal atresia/stenosis (62% vs. 70%) and a depressed nasal bridge. A remarkable difference is the cleft palate, present only in LRS, and dental anomalies (in 54% of NLRS vs. 3% in LRS), which were only described in two lethal cases: one with congenital teeth, and one 2 y/o with features such as amelogenesis imperfecta Type IC (MIM 204690), similar to those caused by *FAM20A* variants.

There are no differences in brain calcifications, but LRS have more structural alterations and NLRS include more deformative brain defects (25 vs. 3%), such as hydrocephalus and cerebellum deformities, which are probably due to space restriction by abnormal bone growth. Further, NLRS are richer in functional neurologic defects, including seizures (17%), delayed development/intellectual disabilities (34%), hypoacusia (30%) and visual defects. Even though many of these features are not reported in LRS, most of them are evident with age as the central nervous system matures. Since some patients with brain calcification have a normal cognitive function and others have cognitive defects in the absence of structural defects, FAM20C deficiency is involved in neurological structural and functional defects.

Among bone features, localized sclerosis is more frequent in NLRS (47%) and can be exquisitely localized (spine sclerosis or femur osteonecrosis), and fracture and pseudo-fractures are described only in LRS. Pectus deformities are present in 10% of NLRS, but are also associated with age. Other extra-craniofacial features with low frequency in NLRS are a small thorax (7%) and respiratory alterations (23%). In contrast, lethal RS present lung hypoplasia in 59% and a small or narrow thorax in 38%, which are not reported in NLRS. Regarding soft tissue calcification defects, the kidney, liver, lung and ovaries can have calcifications. In addition, structural defects in other organs are also present, including renal and urinary defects (hydronephrosis, double renal pelvis and ureters), adrenal gland hyperplasia, thymus hyperplasia, cardiomegaly and absence of pancreatic islets of Langerhans.

## 10. Conclusions

The effect of abnormal FAM20C function is associated with mislocalization or kinase domain loss of function. In LRS, most variants are localized in the kinase domain. Biological effects of FAM20C deficiency are due to hypophosphorylation of its targets, which are more than 100. Although the RS biological approach is related to FAM20C targets involved in mineralization, multiple new targets with potential effects on other organs have been recently described and some could be involved in RS biology. These new targets have important functions, mainly in the brain and heart, which should be considered in RS pathogenesis, as new targets with functions in other organs will surely be identified. FAM20C pathogenic variants can be localized in any of its exons, which can be of different types, and there is no correlation between phenotype and genotype. However, variants producing mislocalization, lack of secretion and kinase activity, could have a greater effect on lethal or severe cases. The clinical distinction between lethal and NLRS is clear in most cases. Clinically, LRS is easily diagnosed because of consistent clinical features, such as ocular proptosis, nasal hypoplasia, choanal atresia or stenosis, osteosclerosis and periosteal reaction. On the other hand, NLRS has a wide spectral phenotype ranging from typical milder features and dental phenotypes, to, less frequently, intellectual disability or developmental delay, seizures, hypoacusia and bone necrosis. The clinical diagnosis of NLRS a challenge due to this variability. There are 70 RS cases described to date—40 LRS and 30 NLRS—and there are likely to be unreported cases, due to early deaths in the former. We believe that a metabolic bone work-up and identification of FAM20C variants should be considered in patients with mineral density defects, mild facial features or teeth defects without common causes. Due to inexpensive and more accessible novel diagnostic techniques, such as massive sequencing, more NLRS cases will probably be soon identified, and new classifications and approaches will be proposed.

## Figures and Tables

**Table 1:** Novel FAM20C targets involved in different functions.

TARGET	General Function	Expression Organ/Site	FAM20C Phosphosites	FAM20C Phosphorylation Effect	Associated Diseases	Ref.
Fibronectin	Major component of the extracellular matrix.	Extracellular matrix.	Ser757, Ser2341, Ser2384			[100,101]
Fibrinogen	Major component of the extracellular matrix.	Liver production and delivered to circulation.	Ser45, Ser56, Ser364, Ser524, S560, Ser609 (alpha chain) Ser68 (gamma chain)		Bleeding and thrombotic disorders.	[13]
Ero1α	Thiol disulfide oxirreductase. Oxidative protein folding, ER proteostasis. ER redox homeostasis. Protection against ER stress.	Majority of cells.	Ser145	Enhances oxidase activity and promotes oxidative protein folding.		[49,102]
wVW	Coagulation. Platelet adhesion and contribution to hemostasis at sites of vascular injury as well as to arterial thrombosis.	Platelets	Ser74, Ser80, Tyr83, Ser93, Ser1517, Ser1613	Enhances platelet adhesion.	von Willebrand disease.	[103]
PCSK9	Cholesterol metabolism. It binds to LDL receptor for its internalization and degradation in endosomes and lysosomes, favoring LDL cholesterol increments.	Liver	Ser47, Ser666, Ser668, Ser688	Enhances LDL receptor degradation, favoring lower LDL cholesterol clearance and its circulatory increase.	GoF variants: Familial Hypercholesterolemia.	[104]
PCSK7	Lipid metabolism. It binds to ApoA-V (an activator of triglycerides clearance) to enhance its degradation in ER and lysosomes, indirectly lowering lipoprotein lipase activity for triglyceride clearance.	Ubiquos, but primary in liver.	Ser505 (Arg504His)	Lower degradation of apoA-V with an increase in circulation, resulting in lower blood levels and higher triglyceride uptake into adipocytes.	LoF variants: lower triglyceride levels. GoF variants: higher levels of PC7 protein and triglyceride levels.	[105]
Sortilin	Protein trafficking in the exocytic and endocytic pathways involved in neuronal viability and glucose homeostasis.	Nervous system, heart and other tissues. SHH regulation.	Ser825	Vascular calcification promotion through higher TNAP activity. Intracellular sorting receptor for apoB and other proteins (Ser825).	Cardiovascular disease.	[106]
HRC	Regulation of calcium sequestration or release in the SR (storage, uptake and release), through interaction with SERCA2a, triadin and RyR2.	Heart, striated and arteriolar smooth muscle.	Ser96	Calcium regulation.	Malignant arrhythmia.	[107]
Calsequestrin-2	Regulation of calcium.	Heart and other tissues.	Ser385	ER/SR calcium homeostasis and cardiac pathophysiology.	Arrhythmia.	[99]
STIM1	Regulation of calcium.	Heart and other tissues.	Ser88	ER/SR calcium homeostasis and cardiac pathophysiology.	Immunodeficiency 10 (MIM 612783) Myopathy, tubular aggregate 1 (MIM160565) Stormorken syndrome (MIM 185070).	[99]

SR, sarcoplasmic reticulum; ER, endoplasmic reticulum; GoF, gain of function; LoF: loss of function.

**Table 2:** Pathogenic FAM20C variants in lethal and non-lethal RS.

Number	Localization	c.Description	p.Description	DNA Type	Protein Type	KD Effect	KD LOCALIZATION	Lethality	Reference
1	7pter	45, XY psudic (7;7) (p22;p22)	-	GR	A	+		+	Simpson et al., 2007 [1]
2	48Kb	46,XY.ar [hg19] 7p22.3 (36480-523731)x0	-	GR	A	+		+	Ababneh et al., 2013 [137]
3	E1	c.456delC	p.Gly153Alafs*34	Del/FS	SP	+		+	El-Dessouky et al., 2020 [138]
4	E1	c.456dupC	Gly153Argfs*56	Dup/FS	SP	+		+	Hernández-Zavala et al., 2020 [139]
5	E1	c.474delC	Ser159Profs*28	Del/FS	SP	+		+	Hernández-Zavala et al., 2020 [139]
6	E1	c.496G>T	p.Glu166*	Su	S	+		-	Mameli et al., 2020 [140]
7	E2	c.784+ 5G>C	p.W202Cfs*37	Su	S/SP	+		-	Acevedo et al., 22015 [141]
8	E2	c.676T>A	p.Trp226Arg	Su	M	-		-	Elalaoui et al., 2016 [142]
9	E2	c.773T>A	p.IIe258Asn	Su	M	-		-	Simpson et al., 2019 [136]
10	E3	c.803C>T	p.Thr268Met	Su	M	-		-	Rafaelsen et al., 2013 [143]
11	E3	c.838 G>A	p.Gly280Arg	Su	M	-		-	Simpson et al., 2009 [136]
12	E3/I	c.863+5G>C	-	Su	S	-		+	Mehme E et al., 2020 [35]
13	E4	c.906C>A	p.Phe302Leu	Su	M	-		-	Rolvien et al., 2018 [5]
14	E4	c.905delT	p.Phe302Serfs*35	Del/FS	SP	+		+	El-Dessouky et al., 2020 [138]
15	E4	c.915C>A	p.Tyr305*	Su	NS	+		-	Rafaelsen et al., 2013 [143]
16	E4	c.940C>T	p.Pro314Ser	Su	M	-		-	Fradin et al., 2011 [144]
17	E4/I4	c.952_956+30dup (+)	-	Dup	S/SP	-		-	Rolvien et al., 2018 [5]
18	E4	c.956G>A	p.Arg319Lys	Su	M			+	Boissel et al., 2017 [145]
19	E4/I4	c.956+5G>C	-	Su	SP	-		+	Simpson et al., 2007 [1]
20	I4/E5	c.957-3C>G	-	Su	SP	-		+	Simpson et al., 2007 [1]
21	E5	c.1007T>G	p.Met336Arg	Su	M	-		+	Hung et al., 2019 [146]
22	E6	c.1094G>A	Gly365Asp	Su	M	+	+	+	Whyte et al., 2016 [135]
23	E6	c.1107_1108insTACTG	p.Tyr369 fs	Ins	FS	+	+	-	Mamedova et al., 2019 [147]
24	E6	c.1135G>A	p.Gly379Arg	Su	M	+	+	+ -	Simpson et al., 2007 [1] Mahmood et al., 2014 [148]
25	E6	c.1136G>A	p.Gly379Glu	Su	M	+	+	+	Simpson et al., 2007 [1]
26	E6	c.1163T>G	p.Leu388Arg	Su	M	+	+	+	Simpson et al., 2007 [1]
27	E6	c.1219T>G	p.Tyr407Gly	Su	M	+	+	-	Tamai et al., 2017 [4]
28	E6	c.1222C>T	p.Arg408Trp	Su	M	+	+	-	Takeyari et al., 2014 [83]
29	E6	c.1225C>T	p.Arg409Cys	Su	M	+	+	+	Seidahmed et al., 2015 [149]
30	E6	c.1228 T>A	p.Ser410Thr	Su	M	+	+	-	Sheth et al., 2018 [6]
31	E7	c.1255T>C	p.Trp419Arg	Su	M	+	+	-	Eras et al., 2021 [150]
32	E7	c.1351G>A	p.Asp451Asn	Su	M	+	+	-	Simpson et al., 2009 [136]
33	E7	c.1351G>A	p.Asp451Asn	Su	M	+	+	-	Mameli et al., 2020 [140]
33	E8	c.1375C>G	p.Arg459Gly	Su	M	+	+	-	Mamedova et al., 2019 [147]
34	I/E8	c.1364-2A>G	-	Su	SP	+	+	+	Simpson et al., 2007 [1]
35	I/E9	c.1446-1G>A	-	Su	SP	+	+	+	Simpson et al., 2007 [1]
36	E9	c.1487C>T	p.Pro496Leu	Su	M	+	+	-	Acevedo et al., 2015 [141]
37	E10	c.1528C>T	p.Arg510Cys	Su	M	+	+	+	Boissel et al., 2017 [145]
38	E10	c.1557C>G	p.Tyr519*	Su	NS	+	+	+	El-Dessouky et al., 2020 [138]
39	E10	c.1645C>T	p.Arg549Trp	Su	M	+	+	+ -	Simpson et al., 2007 [1] Eltan et al., 2020 [151]
40	E10	c.1672C>T	p.Arg558Trp	Su	M	+	+	+	Kochar et al., 2010 [152]
41	E10	c-1680C>A	p.Cys560*	Su	NS	+	+	+	Lulla et al., 2020 [153]

KD, kinase domain; Su, substitution; Del, deletion; Dup, duplication; M, missense; TP, truncated protein; NS, non-sense (STOP); FS, frameshift; A, absence of protein; (+), considered a polymorphism but creates a novel splicing site leading to a premature codon with mild effect.

**Table 3:** Craniofacial features of LRS cases.

Case	Reference	Year	Lethal	Brain Calcifications	Microcephay	Brachycephaly	Wide Fontanelles	Midface Hhypoplasia	Ocular Proptosis	Hypertelorism	Depressed Nasal Bridge	Choanal Stenosis/Atresia	Hypoplastic Nose	Small/Fish-Like Mouth	Macroglosia	Abnormal Teeth	Gum Hyperplasia	Cleft Palate	High Palate	Micrognatia	Low-Set Ears
1	Raine [156]	1989	+		+		+	+	+	+	+		+	+			+	+		+	
2	Kingston et al. [157]	1991	+		+	+	+	+	+		+		+	+			+		+	+	+
3	Kan et al. [158]	1992	+		+		+	+	+		+		+	+			+		+	+	+
4	Patel et al. [7]	1992	+	+	+				+			+									
5	Al Mane et al. [159]	1996	+	+			+	+	+		+	+	+					+		+	+
6	Al Mane et al. 2 [115]	1998	+	+	+		+	+	+		+			+				+		+	
7	Rejjal et al. [160]	1998	+	+			+	+	+		+	+	+					+		+	+
8	Shalev et al. [161]	1999	+	+			+	+	+		+	+	+	+			+	+		+	+
9	Acosta et al. [162]	2000	+			+	+	+	+		+		+	+					+	+	+
10	Mahafza et al. [163]	2001	+				+	+	+		+	+	+				+		+	+	+
11	Hulskamp et al. [164] Ricket et al. [8]	2003–2002	+	+				+	+		+	+	+	+						+	+
12	Hulskamp et al. [164] Ricket et al. [8]	2003–2002	+	+	+	+	+	+	+	+	+	+		+			+	+		+	+
13	Hulskamp et al. [164] Ricket et al. [8] Simpson et al. [1]	2003–2002	+	+	+		+	+	+		+	+	+	+			+			+	+
14	Al-Gazali et al. [165]	2003	+	+			+	+	+		+	+							+		
15	Simpson et al. (1) [1]	2007	+				+	+	+		+										
16	Simpson et al. (3) [1]	2007	+					+	+		+										
17	Simpson et al. (6) [1]	2007	+	+				+	+		+										
18	Simpson et al. (7) [1]	2007	+	+				+	+		+										
19	Chitayat et al. [9]	2007	+	+			+	+	+	+	+		+				+	+	+		
20	Kochar et al. [152]	2010	+	+	+		+	+	+		+	+	+	+			+			+	
21	Michael et al. [10]	2011	+					+	+				+	+			+				
22	Gaigi et al. 1 [166]	2011	+				+	+	+		+	+	+	+			+	+		+	+
23	Gaigi et al. 2 [166]	2011	+				+	+	+		+	+	+	+			+			+	+
24	Gaigi et al. 3 [166]	2011	+					+	+									+			
25	Gaigi et al. 4 [166]	2011	+	+			+	+	+		+	+	+	+			+			+	+
26	Ababneh et al. [137]	2013	+	+			+	+	+		+	+						+		+	+
27	Berger et al. [167]	2014	+	+			+	+	+			+	+	+			+			+	+
28	Seidahmed M et al. [149]	2015	+	+			+	+	+		+	+	+	+					+	+	+
29	Whyte et al. 1 [135]	2016	+	+	+		+	+	+	+	+	+				+				+	+
30	Whyte et al. 2 [135]	2016	+	+	+		+	+	+	+	+	+								+	+
31	Boissel et al. [145]	2017	+	+		+			+		+		+								
32	Hung et al. [146]	2019	+	+	+		+	+	+									+			
33	Hernández-Zavala et al. 1 [139]	2020	+	+	+	+	+	+	+	+	+	+	+				+		+	+	+
34	Hernández-Zavala et al. 2 [139]	2020	+	+		+	+	+	+		+						+		+	+	
35	Eltan et al. [151]	2020	+	+			+	+	+	+		+	+	+	+		+		+	+	
36	El-Dessouky et al. 1 [138]	2020	+	+	+			+	+	+	+	+		+					+	+	+
37	El-Dessouky et al. 2 [138]	2020	+	+	+			+	+	+	+	+		+					+	+	+
38	El-Dessouky et a. 3 [138]	2020	+	+	+			+	+	+	+	+		+					+	+	+
39	Lulla et al. [153]	2020	+	+	+		+	+	+	+	+	+	+	+			+	+	+	+	+
40	Bajaj et al. [154]	2021	+				+	+	+		+	+	+	+		+	+		+	+	

**Table 4:** Skeletal features of LRS cases.

Case	Reference	Year	Lethal	Age/Survival	Death Cause	Population	Consanguinity	Gender	Gestational Weeks	Weight/Percentile	Length/Percentle	Local Osteosclerosis	Generalized Osteosclerosis	Head Circumference/Percentile Circunference/Percentile	Short Neck	Cervical Ossification Defects	Vertebral Defects	Sacral Ossification Defects	Small/Narrow Thorax	Periosteal Reaction	Poor Cortico-Medullary-Differentiation	Bone-in-Bone	Bowed Bones	Fractures	Pseudo-Fractures	Short Limbs	Rhizomelic Shortening	Long Bone Shortening	Brachydactyly	Distal Phalangeal Hypoplasia	Tapered Fingers
1	Raine et al. [156]	1989	+	1	R	Au		F	T	N	<3		+	<3						+											
2	Kingston et al. [157]/Simpson et al. (2) [1]	1991/2007	+	1	R	Cau	+	M	37	N	N		+	3	+				+	+				+					+	+	+
3	Kan et al. [158]	1992	+	1	R			F	37	<3	<3		+	<3					+	+					+						
4	Patel et al. [7]	1992	+		R	SA	+	M	36			+							+												
5	Al Mane et al. [159]	1996	+	4	R	SA	+	M	39	N			+							+	+				+						
6	Al Mane et al. [115]	1998	+			SA	+	F	37	N			+							+					+						
7	Rejjal et al. [160]	1998	+	2	R	SA	+	M		N	N		+	N					+	+											
8	Shalev et al. [161]	1999	+	3	R	SA	+	F	39	N	N		+	N			+		+	+						+					
9	Acosta et al. [162]	2000	+	1		Br	+	M	34	3	<3	+			+					+											+
10	Mahafza et al. [163]	2001	+	3	CR	Jo	+	M	40	N	N		+	N	+				+	+											+
11	Hulskamp et al. (3)/Ricket et al. (1) [8,164]	2003/2002	+	1	R	T	+	M	33	N	N		+	N																	
12	Hulskamp et al. (2)/Ricket et al. (2) [8,164]	2003/2002/2007	+	1	R	T	+	M	24	N	N		+		+				+	+			+						+	+	
13	Hulskamp et al. (1) Ricket et al. (3)/Simpson et al. (5) [1,8,164]	2003–2002	+	2	R	T	+	F	32	N	N		+	N	+				+	+									+		
14	Al-Gazali et al. [1,165]/Simpson et al. (4)	2003	+	4	R	E		M	36	N	N	+		N	+				+	+	+		+			+		+	+		
15	Simpson et al. (1) [1]	2007	+	1	R			M	37				+		+				+	+											
16	Simpson et al. (3) [1]	2007	+				+	M	38			+							+	+											
17	Simpson et al. (6) [1]	2007	+	3				F	38				+							+											
18	Simpson et al. [1]	2007	+					F	32			+							+	+											
19	Chitayat et al. [9]	2007	+	3		Jw		M					+																		
20	Kochar et al. [152]	2010	+	4	PN	I	+	M	40	N	N		+	<3					+	+			+								
21	Michael et al. [10]	2011	+	1		Su	+		34																						
22	Gaigi et al. 1 [166]	2011	+	1	R			M	39	<3	<3		+	<3					+							+					
23	Gaigi et al. 2 [166]	2011	+	1	R			M	42				+																		
24	Gaigi et al. 3 [166]	2011	+	2	R			F	40	<3	<3	+		<3																	
25	Gaigi et al. 4 [166]	2011	+	2				F																							
26	Ababneh et al. [137]	2013	+	4	PN	SA	+	M	T	N			+		+				+	+						+			+	+	
27	Abu et al. [167]	2014	+	4	R	P		M	31	N	N		+	N						+						+					
28	Seidahmed M et al. [149]	2015	+	1	R	SA	+	M	34	N	N		+	N	+		+	+	+											+	
29	Whyte et al. 1 [135]	2016	+	2	R	Cau		F		<3			+	<3					+	+		+									
30	Whyte et al. 2 [135]	2016	+	3	R	Cau		F	37	<3			+						+	+											
31	Boissel et al. 2017 [145]		+	25	SB			F	25				+				+		+												
32	Hung et al. [146]	2019	+	1	R	E	+	M	37	-	-	+		+	+					+				+							
33	Hernández-Zavala et al. [139]	2020	+	2	R	M		M	40	N	N	+	+	N					+	+									+	+	
34	Hernández-Zavala et al. [139]	2020	+	1	R	M		M	21	N	N	+		N					+						+				+	+	
35	Eltan et al. [151]	2020	+	7	R	T	-	M	37	N	N		+	N	+				+	+		+		+	+					+	
36	El-Dessouky et al. 1 [138]	2020	+	1	R	E	+	M	26	N	N			N					+				+	+	+		+				
37	El-Dessouky et al. 2 [138]	2020	+	1	R	E	+	F	38	N	N			N					+								+				
38	El-Dessouky et a. 3 [138]	2020	+	1	R	E		M	38	N	N			N					+					+	+	+	+				
39	Lulla et al. [153]	2020	+			I		F					+						+					+	+						
40	Bajaj et al. [154]	2021	+			I		F	N	N	N		+	N							+			+							

R, respiratory; T, term; SB, stillbirth; F, female; M, male. N, normal; 1, 0–24 h; 2, 2–7 days; 3, 8–30 days; 4, 1–3 months; 5, 4–6 months; 6, 7–12 months; 7, 13–24 months; Au, Australia; Cau, caucasic population; SA, Saudi Arabia; Br, Brazil; Jo, Jordan; T, Turkey; Jw, Jewish; I, India; Su, Sudan; P, Pakistan; E, Ecuador; M, Mexico; E, Egypt. Empty cell: not described or negative feature.

**Table 5:** Craniofacial features of non-lethal lethal RS.

Case	Reference	Year	Non-Lethal	Brain Calcifications	Microcephaly	Brachy/Turri/Plagio-Cephaly	Wide Fontanelles	Bossed Forehead	Midface Hypoplasia	Ocular Proptosis	Hypertelorism	Visual Alterations	Low/Depressed Nasal Bridge	Short/Hypoplastic Nose	Choanal Stenosis/Atresia	Gingival Hyperplasia	High Palate	Large/Protruding Tongue	Abnormal Teeth	Micrognatia	Prognathism	Low Set Ears	Dysplastic Ears	Hypoacusia
1	Simpson et al. [136]	2009	+	+		+				+			+	+		+	+	+	+			+		+
2	Simpson et al. [136]	2009	+	+		+	+		+	+		+	+	+				+				+		+
3	Fradin et al. (1) [144]	2011	+	+							+			+	+		+	+	+					
4	Fradin et al. (2) [144]	2011	+	+		+			+						+					+				
5	koob et al. [11]	2011	+	+					+	+					+			+		+				
6	Rafaelsen et al. (1) [143]	2013	+	+					+				+		+				+	+		+		
7	Rafaelsen et al. (2) [143]	2013	+	+					+				+		+				+			+		
8	Vishwanath et al. [171]	2014	+	+	+		+			+			+	+	+		+			+			+	
9	Mahmood et al. [148]	2014	+	+	+		+		+	+	+		+	+	+							+		
10	Takeyari et al. [83]	2014	+	+																				
11	Acevedo et al. (1) [141]	2015	+		+				+	+					+		+		+	+			+	+
12	Acevedo et al. (2) [141]	2015	+		+				+	+							+		+	+			+	+
13	Acevedo et al. (3) [141]	2015	+	+					+	+							+		+	+			+	+
14	Acevedo et al. (4) [141]	2015	+	+	+				+	+		+	+	+	+	+	+		+	+		+		
15	Acevedo et al. (5) [141]	2015	+	+	+				+	+			+	+		+	+		+	+		+		
16	Elalaoui et al. (1) [142]	2016	+	+					+		+		+				+		+		+	+		+
17	Elalaoui et al. (2) [142]	2016	+						+		+		+				+	+	+		+	+		+
18	Das et al. [172]	2017	+	+			+			+			+		+		+					+		
19	Tamai et al. [4]	2017	+	+					+	+	+		+	+	+					+				+
20	Sheth et al. [6]	2018	+		+						+		+											
21	Rolvien et al. [5]	2018	+																					
22	Mamedova et al. [147]	2019	+	+					+	+	+			+					+			+		
23	Mameli et al. 1 [140]	2020	+	+	+		+			+	+					-			+			-		+
24	Mameli et al. 2 [140]	2020	+	+	+				+	+			+		+									
25	Mameli et al. 3 [140]	2020	+	-	+				+	+	+				+	+			+			+		
26	Günes et al. [173]	2005	+	+	+		+		+	+	+		+	+			+	+		+		+		
27	Hirst et al. 1 [174]	2021	+						+	+							+		+					
28	Hirst et al. 2 [174]	2021	+	+		+		+	+		hyp					+	+		+					
29	Hirst et al. 3 [174]	2021	+	+					+	+				+			+		+					
30	Eras et al. [150]	2021	+	+					+	+			+	+	+									+

Hyp: hypotelorism. Empty cell: Not described or negative feature.

**Table 6:** Skeletal abnormalities in non-lethal Raine syndrome.

Case	Reference	Year	Non-Lethal	Age (Years)	Population	Consanguinity	Gender	Gestational Weeks	Length (Percentile)	Local Osteosclerosis	Generalized Osteosclerosis	Head Circumference	Craniosynostosis	Small Thorax	Pectus Excavatum	Cervical Ossification Defects	Vertebral Segmentation Defects	Sacral Ossification Defects	Periosteal Reaction	Short Limbs	Brachydactyily	Fingerpads	Thick Fingers	Fractures/Pseudofractures	Poor Cortico-Medullary-Differentiation
1	Simpson et al., 1 [136]	2009	+	8y	ND	+	M	38			+														
2	Simpson et al., 2 [136]	2009	+	11y	ND	+					+				+							+	+		
3	Fradin et al., 1 [144]	2011	+	4y	ND	+		37	N	+		N													
4	Fradin et al., 2 [144]	2011	+	1y	Al	+		38	<3		+	N		+		+	+	+			+				
5	Koob et al. [11]	2011	+	1y	Al	+	F	N			+			+		+		+			+				
6	Rafaelsen et al., 1 [143]	2013	+	18y	Nw		M	36	N	+		N											+		
7	Rafaelsen et al., 2 [143]	2013	+	16y	Nw		M	37	N	+		N													
8	Vishwanath et al. [171]	2014	+	1mo	I	+			<P3		+	<P3				+					+				
9	Mahmood et al. [148]	2014	+	3y	As	+	M	36	N		+									+					
10	Takeyari et al. [83]	2014	+	61y	Jp	+	M		<3	+									+						
11	Acevedo et al., 1 [141]	2015	+	27y	Br	+	M			+															
12	Acevedo et al., 2 [141]	2015	+	22y	Br	+	M			+											+				
13	Acevedo et al., 3 [141]	2015	+	21y	Br	+	F			+															
14	Acevedo et al., 4 [141]	2015	+	5y	Br	+	M															+			
15	Acevedo et al., 5 [141]	2015	+	4y	Br	+	M															+			
16	Elalaoui et al., 1 [142]	2016	+	18y	Mor	+	F	40	N											+	+	+			
17	Elalaoui et al., 2 [142]	2016	+	15y	Mor	+	M	40	N												+				
18	Das et al. [172]	2017	+	3mo	ND		F				+														
19	Tamai et al. [4]	2017	+	2y	Jp			38	<3	+		N													
20	Sheth et al. [6]	2018	+	6y	I	+	F	40		+															
21	Rolvien et al. [5]	2018	+	72y	ND					+															
22	Mamedova et al. [147]	2019	+	39y	Ar		F			+					+				+		+				
23	Mameli et al., 1 [140]	2020	+	12y	P		F				+	<3													
24	Mameli et al., 2 [140]	2020	+	5mo	P		M	37	N		+	<3													
25	Mameli et al., 3 [140]	2020	+	4mo	P		M	37	N		+	<3													
26	Günes et al. [173]	2005	+	2hr	T	+	f	ND	N		+	<10							+						
27	Hirst et al., 1 [174]	2021	+	14	So		M				+									+					
28	Hirst et al., 2 [174]	2021	+	13y	So		M				+														
29	Hirst et al., 3 [174]	2021	+	11y	So		F		<3		+		+												
30	Eras et al. [150]	2021	+	1y	ND	+	M	40	N		+	N										+			

y, years; mo, months; Al, Algeria; Nw, Norway; I, India; As, Asia; Jp, Japan; Br, Brazil; Mor, Morocco; Ar, Armenia; P, Pakistan; T, Turkey; So, Somalia; M, male; F, female; N, normal; ND, not described. Empty cell: Not described or negative feature.

**Table 7:** Neurological defects in Raine syndrome.

Feature Description	LC	NLC	References (NLC)	References (LC)
Developmental delay/disability, Language delay		14	[6,136,140,141,142,143]	
Seizures		8	[136,141,142,148,150]	
Hypoacusia		10	[4,136,140,141,142,150]	
Hydrocephaly		3	[136,148]	
Visual impairment		2	[140,141]	
Dystonic movements		1	[148]	
Hypertonia		1	[148]	
Hypotonia	3	1	[6]	[135,137]
Non-visualization of pituitary gland		1	[172]	
Mild cortical atrophy		1	[6]	
Hypoplastic appearance of posterior part of the brain		1	[6]	
Corpus callosal (agenesia, hypoplasia)	2	1	[6]	[139,151]
Mild paucity of the peritrigonal white matter surrounding the trigones of bilateral lateral ventricles		1	[6]	
Cerebellum (conical, hypoplasia)	3			[9,149,158]
Cortical defects (abnormal gyral pattern, dysplasia, disorganization of the cortical layers)	3			[9,139,149]
Gliosis/astrogliosis	2			[158]
Encephalocele	2			[115,146]
Posterior fossa (small or hypoplastic)	2			[9,151]
Optic nerves and chiasm (small, atrophy)	2			[158,161]
Choroid plexus (bilateral cysts)	1			[10]
Cerebral atrophy	1			[151]
Hydrocephaly	1			[151]
Microphtalmia	1			[151]

LC, lethal cases; NLC, non-lethal cases.

## Data Availability

Not applicable.
